# New 8-prenylated quercetin glycosides from the flowers of *Epimedium acuminatum* and their testosterone production-promoting activities

**DOI:** 10.3389/fchem.2022.1014110

**Published:** 2022-10-10

**Authors:** Yixin Zhang, Cheng Zhang, Zihan Li, Cheng Zeng, Zhen Xue, Erwei Li, Gang Li, Juan Li, Guoan Shen, Chaoqun Xu, Yuanyue Wang, Baiping Ma, Hui Zhang, Baolin Guo

**Affiliations:** ^1^ Key Laboratory of Bioactive Substances and Resources Utilization of Chinese Herbal Medicines, Ministry of Education, Institute of Medicinal Plant Development, Chinese Academy of Medical Sciences and Peking Union Medical College, Beijing, China; ^2^ Key Laboratory of Biodiversity Science and Ecological Engineering, Ministry of Education, College of Life Sciences, Beijing Normal University, Beijing, China; ^3^ Hubei Province Key Laboratory of Traditional Chinese Medicine Resource and Chemistry, Hubei University of Chinese Medicine, Wuhan, Hubei, China; ^4^ Key Laboratory of Plant Molecular Physiology, Institute of Botany, Chinese Academy of Sciences, Beijing, China; ^5^ State Key Laboratory of Mycology, Institute of Microbiology, Chinese Academy of Sciences, Beijing, China; ^6^ Department of Pharmaceutical Sciences, Beijing Institute of Radiation Medicine, Beijing, China

**Keywords:** *Epimedium* flavonols, phytochemical investigation, flowers, testosterone production, structure-activity relationship, *Epimedium acuminatum*

## Abstract

Phytochemical investigation was carried out for the flowers of *Epimedium acuminatum* Franchet. by first conducting LC-MS analysis, leading to the identification of 32 compounds. Furthermore, guided by LC-MS profiling, three new 8-prenylated quercetin glycosides (3′-hydroxylikarisoside C, 3′-hydroxylepimedoside E, 3′-hydroxyldiphylloside B), one new anthocyanin (delphinidin-3-*O*-*p*-coumaroyl-sophoroside) and six known compounds were isolated from the flowers of *E. acuminatum* for the first time, and their structures were characterized based on spectroscopic methods including 1D and 2D NMR, and HRESIMS. Combining our discoveries and literature survey, a revised classification of *Epimedium* flavonols was proposed as Type A (8-prenylated kaempferol based), which was further subdivided into subtype icaritin and subtype demethylicaritin, and Type B (8-prenylated quercetin based), which was further subdivided into subtype 3′-hydroxylicaritin and subtype 3′-hydroxyldemethylicaritin. The structure-activity relationship (SAR) study was carried out by comparing testosterone production-promoting activities of all the new compounds along with nine related *Epimedium* flavonols, revealing that the new 8-prenylated quercetin glycosides (subtype 3′-hydroxyldemethylicaritin in Type B) exhibited lower testosterone production-promoting activities in rat primary Leydig cells than *Epimedium* flavonols of subtype demethylicaritin in Type A, but possessed higher activities than the *Epimedium* flavonols of subtype icaritin in Type A. These results suggested that either methylation at C-4′ position or hydroxylation at C-3′ position of ring B could significantly reduce the testosterone production-promoting activities of *Epimedium* flavonols.

## Graphical Abstract

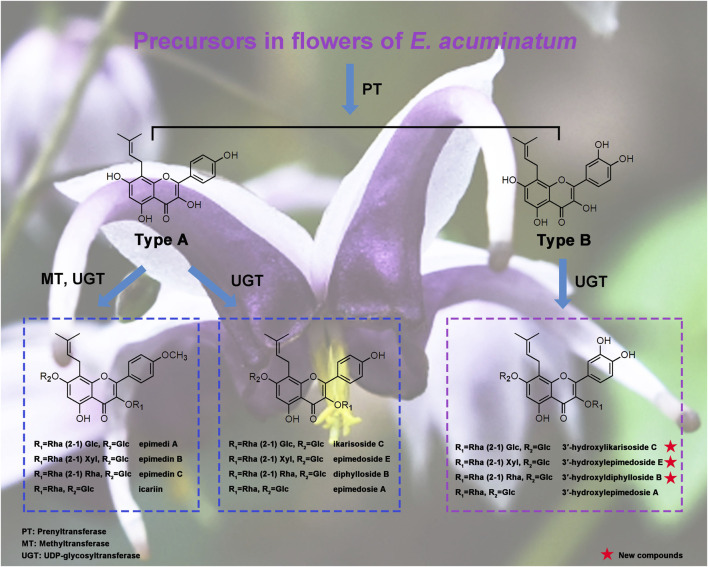

## Introduction

*Epimedium* L. is a medicinally important herbaceous genus in the family Berberidaceae containing more than 60 perennial plant species. The native occurrences of these plants are geographically discontinuous from North Africa (Algeria) to East Asia, with most of them endemic to China (Stearn, 2002; Ying, 2002). Due to their special nourishing effects on kidney and bones, the leaves and rhizomes of *Epimedium* plants have long been used in traditional Chinese medicine (GMP, 2019; CP, 2020). Meanwhile, bearing beautiful evergreen heart-shaped leaves, and magnificent “spider-like” flowers, *Epimedium* plants are popular as garden plants for groundcover and ornamental decoration in Europe, North America and Japan (usually known as barrenworts) (Ma et al., 2011; Lone et al., 2018). Among *Epimedium* species, the leaves and rhizomes of *Epimedium acuminatum* Franch. were recorded as “HERBA EPIMEDII ACUMINATI” and “EPIMEDII RHIZOMA ET RADIX,” respectively, in the Quality standard of Ethnic Chinese medicinal materials in Guizhou province (GMP, 2003; GMP, 2019). Besides, its wide distribution in the southeastern part of China and attractive purplish red flowers have endowed it with great cultivation prospects, and therefore was selected for current study.

It was first in ancient Chinese materia medica “*Shennong Bencao Jing* (*Sheng Nong’s herbal classic*)” that *Epimedium* herbs were documented with main effects of treating sexual dysfunction, tonifying kidney Yang, bones and strength. Nowadays, modern pharmacological studies have verified a specific class of 8-prenylated flavonols in *Epimedium* plants as the major bioactive components (*Epimedium* flavonols), which exhibit extensive bioactivities of regulating hormone production, modulating immunological function, anti-rheumatic, anti-osteoporosis, anti-cancer, anti-aging, etc (Wu et al., 2003; Ma et al., 2011).

**FIGURE 1:**
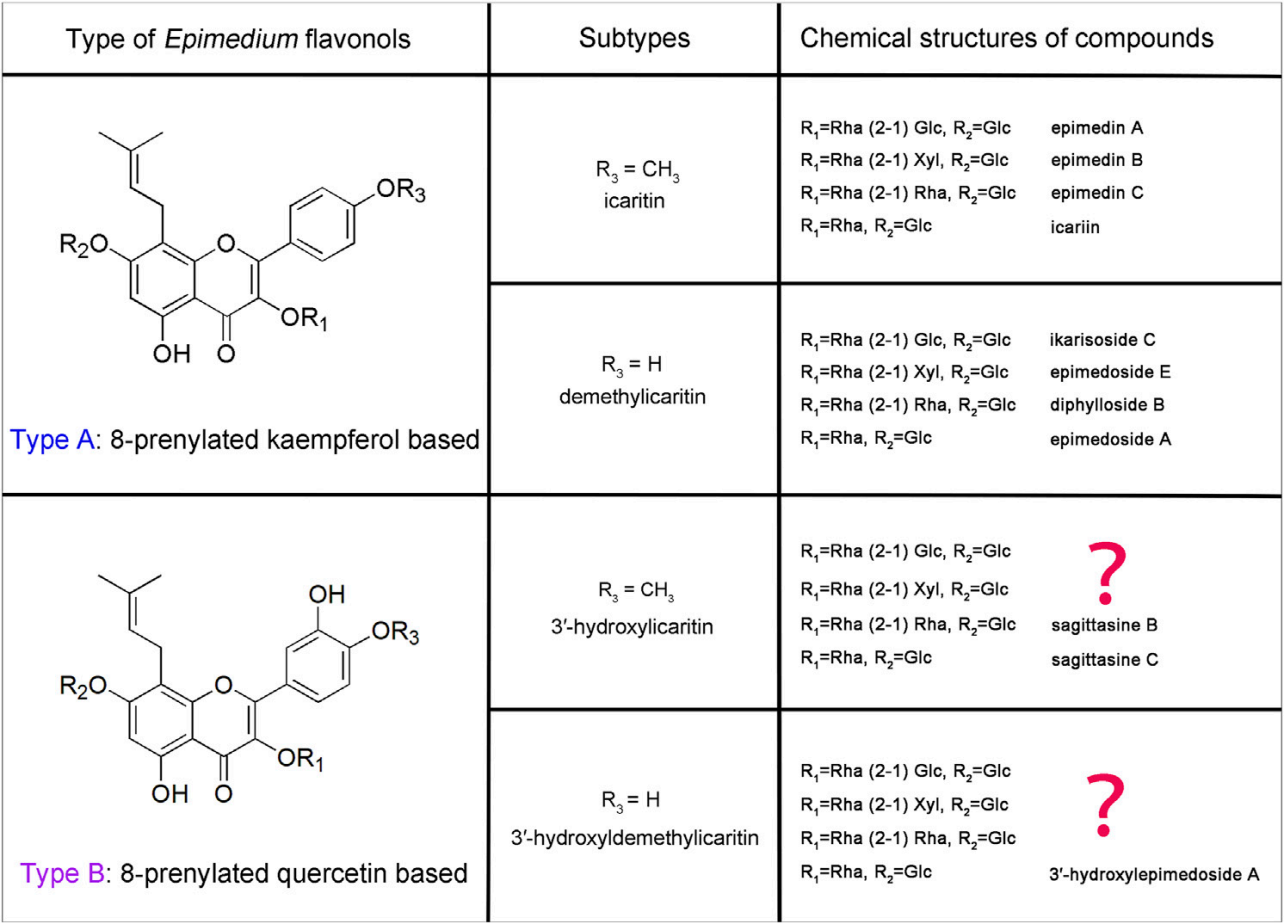
Classification of key *Epimedium* flavonols.

These *Epimedium* flavonols possess basic chemical structures with a prenyl group at C-8 position of ring A, and with the most common glycosylation pattern: a glucose at C-7, and a rhamnose initially at C-3 following the addition of glucose, xylose or rhamnose (1–2 linkage). However, the key difference is the presence or absence of hydroxyl group at C-3′ position of ring B. Depicted in Figure 1 are two key types of *Epimedium* flavonols, among which Type A *Epimedium* flavonols with the backbone of 8-prenylated kaempferol are widely found in *Epimedium* species, including subtypes of icaritin (4′-methoxyl) and demethylicaritin (4′-hydroxyl) (Jian et al., 2015; Zhou et al., 2021). By contrast, Type B of *Epimedium* flavonols with the backbone of 8-prenylated quercetin are rarely isolated or detected, including subtypes of 3′-hydroxylicaritin (4′-methoxyl) (Wang et al., 2007; Tu et al., 2011) and 3′-hydroxyldemethylicaritin (4′-hydroxyl) (Li et al., 2017), shown in Figure 1. Based on the common glycosylation pattern of *Epimedium* flavonols, it is rational to speculate the existence of a series of 8-prenylated quercetin glycosides in *Epimedium* species, and the bioactivities of which are worthy to be explored.

Until now, the leaves of *Epimedium* plants have been extensively examined and mainly found to produce *Epimedium* flavonols of icaritin subtype in Type A, and the rhizomes have been proven to principally accumulate *Epimedium* flavonols of demethylicaritin subtype in Type A (Jian et al., 2015; Zhou et al., 2021). By contrast, there has been few phytochemical researches focusing on flowers, leading to the limited understanding on the chemical constituents of *Epimedium* flowers (Yoshitama, 1984; Qin et al., 2022). Since flowers are taxonomically important organs and a potential source for medicinal use, the chemical constituents in different parts of *E. acuminatum* flowers (petals and inner sepals) were thoroughly explored by combining LC-MS and NMR data. Guided by a preliminary LC-MS profiling, three new 8-prenylated quercetin glycosides (subtype 3′-hydroxyldemethylicaritin in type B), one new anthocyanin and six known flavonols were isolated from flowers of *E. acuminatum*, structures of which were determined through 1D and 2D NMR, and HRESIMS. Based on our discoveries and literature survey, a revised classification for *Epimedium* flavonols was proposed. Finally, the testosterone production-promoting activities of all the new compounds were tested and compared with nine related *Epimedium* flavonols, and their structure-activity relationship (SAR) were analyzed, revealing that *Epimedium* flavonols of subtype demethylicaritin in Type A possessed the highest potency for stimulating the testosterone production, and either methylation at C-4′ position or hydroxylation at C-3′ position of ring B could decrease the bioactivity. Combining structural characterization and SAR data, our findings could provide fundamental rules for understanding structural variations in *Epimedium* flavonols, potential candidates for medicinal use, and assist the selection of substrates for identification of prenyltransferases (PTs), UDP-glycosyltransferases (UGTs) and methyltransferases (MTs) in *Epimedium* plants.

## Results

### LC-MS identification of compounds in petals and inner sepals of *E. acuminatum*

**FIGURE 2:**
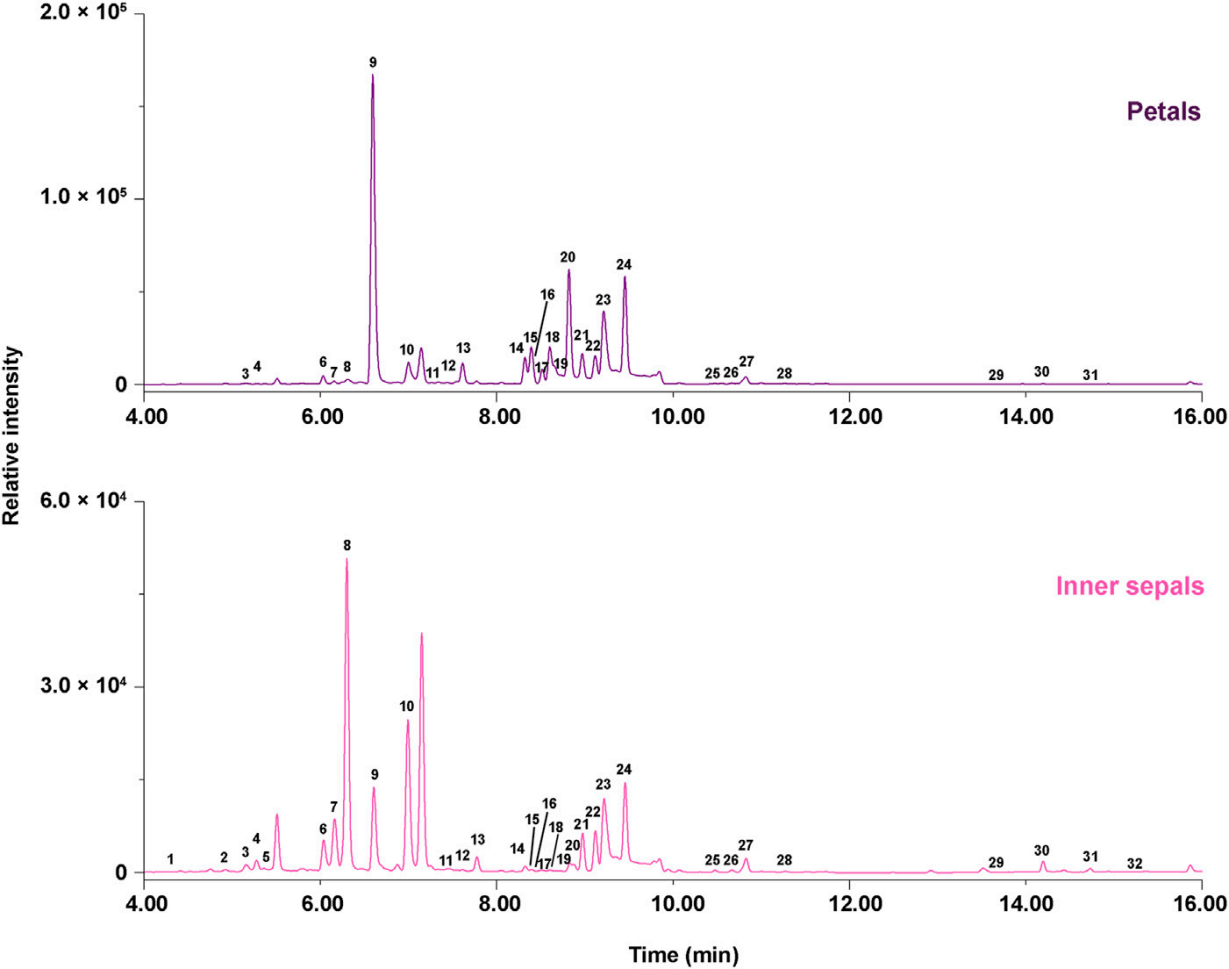
Ultra-high-performance liquid (UPLC) chromatograms at 280 nm for MeOH (0.1% acetic acid) extracts of petals and inner sepals of *E. acuminatum*. Upper panel: petals; Lower panel: inner sepals.

The ultraviolet-visible spectroscopy (UV-Vis spectra) showed the *λ*_max_ at 520 nm for peaks **1**, **3**, **6**, and **9** (Figure 2), which was very close to delphinidin. These peaks exhibited major fragment ions at *m/z* 303, indicating that they were delphinidin-derived anthocyanins. The UV-Vis spectra showed the *λ*_max_ at 520 nm for peaks **2** and **5** (Figure 2), which was very close to petunidin. These compounds exhibited major fragment ions at *m/z* 317, indicating that they were petunidin-derived anthocyanins. The chemical structure of anthocyanins was tentatively identified by comparison with MS/MS data from authentic samples and references (Table 1). Peak **1** was identified as delphinidin-3,5-di-*O*-glucoside for producing molecular ions at *m/z* 627.1577 [M]^+^, and major fragment ions at *m/z* 465.1003 [M-Glc]^+^ and *m/z* 303.0490 [M-Glc-Glc]^+^. Peak **3** was identified as delphinidin-3-*O*-glucoside for giving molecular ions at *m/z* 465.1003 [M]^+^ with fragment ions of *m/z* 303.0490 [M-Glc]^+^, and was confirmed with authentic samples. Peaks **6** produced molecular ions at *m/z* 789.1882 [M]^+^ with only fragment ions at *m/z* 303.0490, indicating an unidentified delphinidin derivative, and was deduced to be delphinidin-3-*O*-caffeoyl-sophoroside. Peak **9** produced molecular ions at *m/z* 773.1935 [M]^+^, with only fragment ions at *m/z* 303.0490, indicating an unidentified delphinidin derivative (further isolated and determined with NMR data). Peak **2** was identified as petunidin-3,5-di-*O*-glucoside for showing molecular ions at *m/z* 641.1736 [M]^+^, and fragment ions at *m/z* 479.1200 [M-Glc]^+^ and *m/z* 317.1170 [M-Glc-Glc]^+^. Peak **5** was identified as petunidin-3-*O*-glucoside for giving molecular ions at *m/z* 479.1200 [M]^+^ and fragment ions at *m/z* 317.1170 [M-Glc]^+^.

**TABLE 1:** Compounds identified in MeOH (0.1% acetic acid) extracts of petals and inner sepals from *E. acuminatum*.

Peak num	Rt	Formula	Precursor ions	Mass	Calc. Mass	Error (mDa)	MS/MS	UVmax	Identification	Existence (petals or sepals)
1	4.27	C_27_H_31_O_17_	[M]^+^	627.1577	627.1561	1.6	465.1199, 303.0490	280, 520	delphinidin-3,5-di-*O*-glucoside	sepals
2	4.87	C_28_H_33_O_17_	[M]^+^	641.1736	641.1718	1.8	479.1200, 317.1170	280, 520	petunidin 3,5-di-*O*-glucoside	sepals
3	5.20	C_21_H_21_O_12_	[M]^+^	465.1003	465.1033	−3.0	303.0490	280, 520	delphinidin-3-*O*-glucoside	petals, sepals
4	5.27	C_16_H_18_O_9_	[M+Na]^+^	377.0835	377.0849	−1.4	197.0273, 173.0791, 163.0383	298, 324	neochlorogenic acid	petals, sepals
5	5.94	C_22_H_23_O_12_	[M]^+^	479.1200	479.1190	1.0	317.1170	280, 520	petunidin-3-*O*-glucoside	sepals
6	6.03	C_36_H_37_O_20_	[M]^+^	789.1882	789.1878	0.4	303.0490	280, 320, 520	delphinidin-3-*O*-caffeoyl-sophoroside	petals, sepals
7	6.15	C_16_H_18_O_9_	[M+Na]^+^	377.0835	377.0849	−1.4	197.0273, 173.0791, 163.0383	298, 324	chlorogenic acid	petals, sepals
8	6.30	C_16_H_18_O_9_	[M+Na]^+^	377.0835	377.0849	−1.4	197.0273,173.0791, 163.0383	298, 324	chlorogenic acid isomer	petals, sepals
9	6.59	C_36_H_37_O_19_	[M]^+^	773.1935	773.1929	0.6	303.0490	280, 320, 520	delphinidin-3-*O*-*p*-coumaroyl-sophoroside (isolated)	petals, sepals
10	7.01	C_20_H_24_NO_4_	[M]^+^	342.1694	342.1705	−1.1	297.1098, 277.0236, 265.0835	268, 301	magnoline	petals, sepals
11	7.61	C_21_H_20_O_13_	[M+H]^+^	481.0978	481.0982	−0.4	319.0431	255, 353	myricetin-3-*O*-glucoside isomer	petals, sepals
12	7.64	C_21_H_20_O_11_	[M+H]^+^	449.1130	449.1084	4.6	303.0490	255, 353	quercetin-3-*O*-rhamnoside isomer	petals, sepals
13	7.73	C_21_H_20_O_10_	[M+H]^+^	433.1120	433.1135	−1.5	287.0540	255, 353	kaempferol-3-*O*-rhamnoside isomer	petals, sepals
14	8.32	C_21_H_20_O_12_	[M+H]^+^	465.1052	465.1033	1.9	303.0490	255, 353	hyperoside (isolated)	petals, sepals
15	8.36	C_21_H_20_O_12_	[M+Na]^+^	487.0857	487.0852	0.5	303.0490	255, 353	isoquercitrin	petals, sepals
16	8.39	C_38_H_48_O_21_	[M+H]^+^	841.2774	841.2766	0.8	679.2239, 533.1674, 371.1117, 315.0514	255, 353	3′-hydroxylikarisoside C (isolated)	petals, sepals
17	8.51	C_37_H_46_O_20_	[M+H]^+^	811.2676	811.2661	1.5	679.2239, 533.1674, 371.1117, 315.0514	255, 353	3′-hydroxylepimedoside E (isolated)	petals, sepals
18	8.65	C_38_H_48_O_20_	[M+H]^+^	825.2831	825.2817	1.4	679.2239, 533.1674, 371.1117, 315.0514	255, 353	3′-hydroxyldiphylloside B (isolated)	petals, sepals
19	8.82	C_21_H_20_O_11_	[M+H]^+^	449.1082	449.1084	−0.2	287.0540	255, 353	astragalin	petals, sepals
20	8.83	C_32_H_38_O_16_	[M+H]^+^	679.2239	679.2238	0.1	533.1674, 371.1117, 315.0514	255, 353	3′-hydroxylepimedoside A (isolated)	petals, sepals
21	8.96	C_38_H_48_O_20_	[M+H]^+^	825.2831	825.2817	1.4	663.2256, 517.1721, 355.1170, 287.0540	268, 322, 360	ikarisoside C (isolated)	petals, sepals
22	9.11	C_37_H_46_O_19_	[M+H]^+^	795.2740	795.2712	2.8	663.2256, 517.1721, 355.1170, 287.0540	268, 322, 360	epimedoside E (isolated)	petals, sepals
23	9.25	C_38_H_48_O_19_	[M+H]^+^	809.2872	809.2868	0.4	663.2256, 517.1721, 355.1170, 287.0540	268, 322, 360	diphylloside B (isolated)	petals, sepals
24	9.46	C_32_H_38_O_15_	[M+H]^+^	663.2256	663.2289	−3.3	517.1721, 355.1170, 287.0540	268, 322, 360	epimedoside A (isolated)	petals, sepals
25	10.49	C_39_H_50_O_20_	[M+H]^+^	839.3002	839.2974	2.8	677.2462, 531.1848, 369.1346, 313.0690	270, 323, 355	epimedin A	petals, sepals
26	10.66	C_38_H_48_O_19_	[M+H]^+^	809.2872	809.2868	0.4	677.2462, 531.1848, 369.1346, 313.0690	270, 323, 355	epimedin B	petals, sepals
27	10.83	C_39_H_50_O_19_	[M+H]^+^	823.3053	823.3025	2.8	677.2462, 531.1848, 369.1346, 313.0690	270, 323, 355	epimedin C	petals, sepals
28	11.27	C_33_H_40_O_15_	[M+H]^+^	677.2462	677.2445	1.7	531.1848, 369.1346, 313.0690		icariin	petals, sepals
29	13.57	C_32_H_38_O_14_	[M+H]^+^	647.2328	647.2340	−1.2	355.1170	268, 322, 360	baohuoside III	petals, sepals
30	14.23	C_26_H_28_O_10_	[M+H]^+^	501.1760	501.1761	−0.1	355.1170	268, 322, 360	baohuoside II	petals, sepals
31	14.70	C_33_H_40_O_14_	[M+H]^+^	661.2480	661.2496	−1.6	515.1937, 369.1346	270, 323, 355	2″-*O*-rhamnosylicariside II	petals, sepals
32	15.31	C_27_H_30_O_10_	[M+H]^+^	515.1937	515.1917	2.0	369.1346	270, 323, 355	baohuoside I	sepals

The UV-Vis spectra showed the *λ*_max_ at around 240−280 nm and 350−360 nm for peaks **11**−**32** (Figure 2), indicating that these peaks corresponded to flavonols. Non-prenylated flavonols constituted the first set of flavanols found in the petals and inner sepals of *E. acuminatum*: peak **11** was identified as myricetin-3-*O*-glucoside isomer with molecular ions at *m/z* 481.0978 [M+H]^+^ and fragment ions at *m/z* 319.0431 [M+H-Glc]^+^; peak **12** was identified as a quercetin-3-*O*-rhamnose isomer giving molecular ions at *m/z* 449.1130 [M+H]^+^ and fragment ions at *m/z* 303.0490 [M+H-Rha]^+^; peak **13** were identified as kaempferol-3-*O*-rhamnose isomer producing molecular ions at *m/z* 433.1120 and fragment ions at *m/z* 287.0540 [M+H-Rha]^+^. By comparison with reference standards, peak **14**, **15**, and **19** were identified as hyperoside, isoquercitrin and astragalin respectively, and their molecular and fragment ions were shown in Table 1.

Peaks **21**−**24** and **25**−**28** (Figure 2) constituted the second set of flavanols (Type A), producing fragment ions at *m/z* 355 and 369, which contain a backbone of 8-prenylated kaempferol. With fragment ions at *m/z* 355 (demethylicaritin), peak **21** was identified as ikarisoside C for giving molecular ions at *m/z* 825.2831 [M+H]^+^, and fragment ions at *m/z* 663.2256 [M+H-Glc]^+^ and *m/z* 517.1721 [M+H-Glc-Rha]^+^; similarly, peak **22**, **23**, and **24** were identified as epimedoside E, diphylloside B, and epimedoside A with molecular ions at *m/z* 795.2740 [M+H]^+^, *m/z* 809.2872 [M+H]^+^, and *m/z* 663.2256 [M+H]^+^ (Table 1). Furthermore, these compounds corresponding to peaks **21**–**24** were directly isolated and structurally confirmed with NMR data. With fragment ions at *m/z* 369 (icaritin), peak **25** was identified as epimedin A for producing molecular ions at *m/z* 839.3002 [M+H]^+^, *m/z* 677.2462 [M+H-Glc]^+^, *m/z* 531.1848 [M+H-Glc-Rha]^+^, and *m/z* 369.1346 [M+H-Rha-Rha-Glc]^+^; similarly, peak **26**, **27** and **28** were identified as epimedin B, epimedin C and icariin, separately, and were confirmed with the authentic samples (Table 1).

Peaks **16**−**18**, and **20** (Figure 2) constituted the third set of flavanols (Type B) with fragment ions at *m/z* 371, indicating a backbone of 8-prenylated quercetin (3′-hydroxyldemethylicaritin). Peaks **16**−**18**, and **20** gave molecular ions at *m/z* 679.2239 [M+H-Glc]^+^ and fragment ions at *m/z* 533.1674 [M+H-Glc-Rha]^+^ (Table 1), and these compounds were directly isolated and structurally confirmed with NMR data (see Section 2.2 and Section 5.4).

### Structural elucidation

**FIGURE 3:**
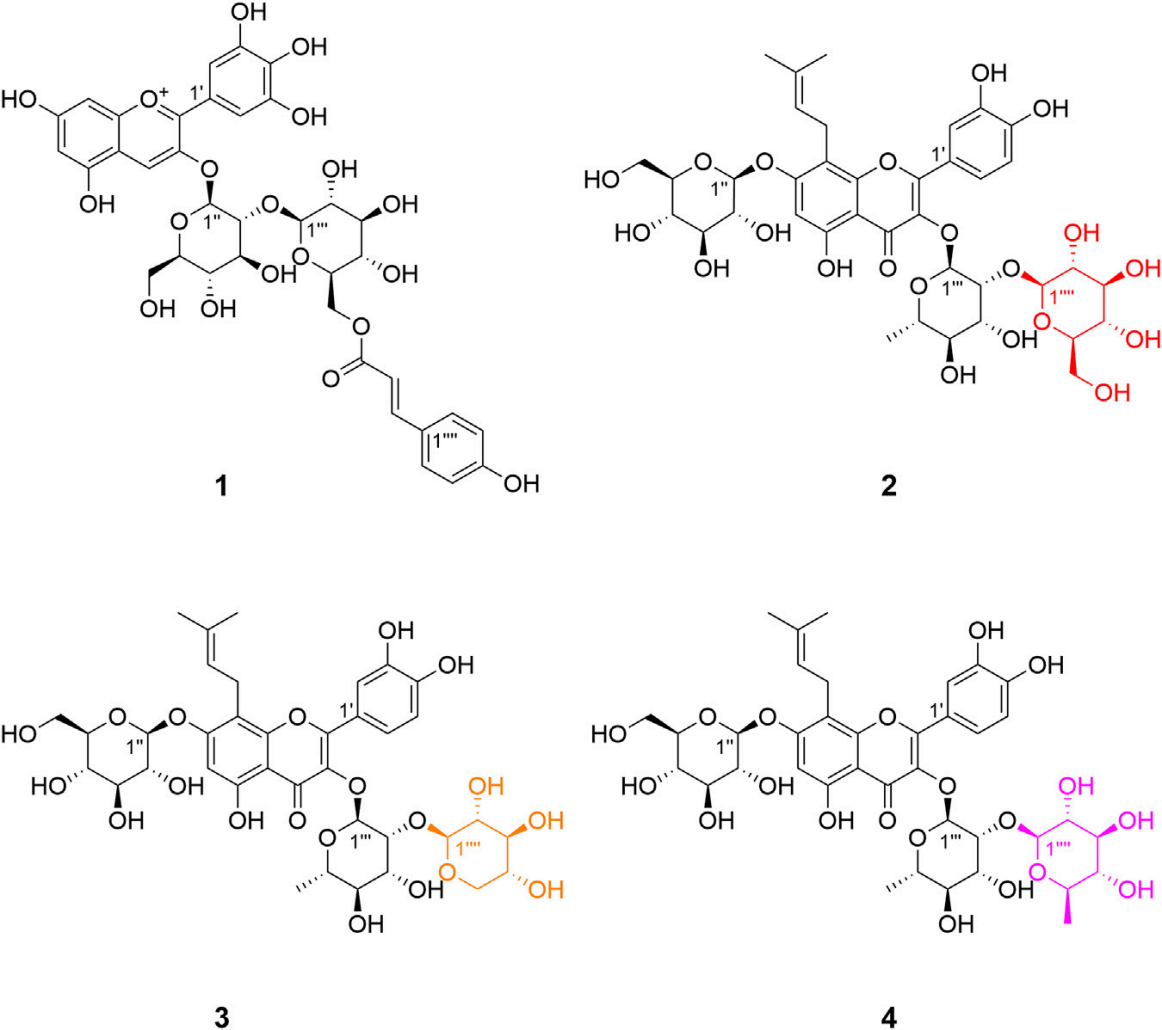
Structures of compounds **1**–**4** isolated from flowers of *E. acuminatum*.

**FIGURE 4:**
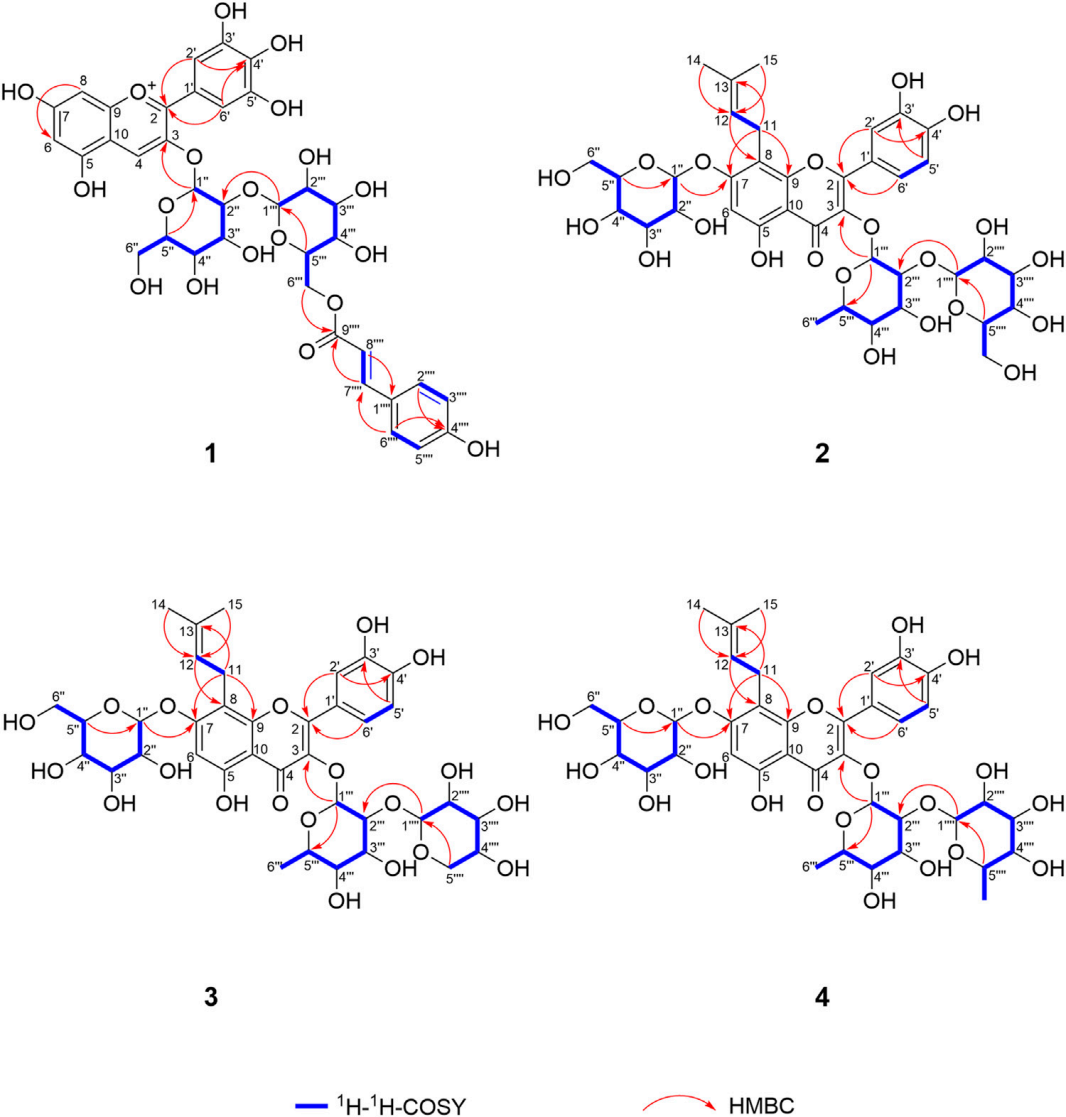
^1^H-^1^H-COSY and key HMBC correlations of compounds **1**–**4**.

Delphinidin 3-*O*-*p*-coumaroyl-sophoroside (**1**, peak **9**) was isolated as dark purplish red powder, the HRESIMS data (*m/z* 773.1940 [M]^+^, calcd for C_36_H_37_O_19_^+^ 773.1929) gave a molecular formula of C_36_H_37_O_19_^+^. The presence of major fragment ion at 303.0482 [M]^+^, in combination with the ^1^H NMR and ^13^C NMR data with characteristic proton chemical shifts shown in Table 2, demonstrated a skeleton of delphinidin. The presence of two sugar moieties was deduced from anomeric hydrogen signals at *δ*_H_ 4.80 (1H, d, *J* = 7.6 Hz) and *δ*_H_ 5.34 (1H, d, *J* = 7.5 Hz), and both were detected to be d-glucose from acid hydrolysis experiments. The *β*-configuration of glucose moieties was assumed by the observed coupling constants (*J* = 7.6 and 7.5 Hz) (Agrawal, 1992). The linkage of Glc A to C-3 of delphinidin, and the Glc A (2→1) Glc B was confirmed by HMBC correlations of H-1″ (*δ*_H_ 5.34) of Glc A and H-1‴ (*δ*_H_ 4.80) of Glc B with C-3 (*δ*_C_ 145.0) and C-2″ (*δ*_C_ 84.1) of Glc A, respectively. Additionally, the aromatic signals of the 1,4-substituted benzene ring (*δ*_H_ 6.72, 2H, d, *J* = 8.6 Hz, and *δ*_H_ 7.07, 2H, d, *J* = 8.6 Hz), and an ester group (*δ*_C_ 168.6), together with two olefinic protons signals exhibiting large coupling constants (*δ*_H_ 5.71, 1H, d, *J* = 16.0 Hz and *δ*_H_ 7.09 1H, d, *J* = 16.0 Hz) indicated a *p*-coumaric acid in *trans*-configuration, which was further confirmed with HMBC correlations from H-2‴′ (*δ*_H_ 7.07) to C-7‴′ (*δ*_C_ 146.3), and from H-7‴′ (*δ*_H_ 7.09) to C-9‴′ (*δ*_C_ 168.6). The acylation of Glc B with *p*-coumaric acid was deduced from HMBC correlations of methylene protons (*δ*_H_ 4.00, H-6a″ and 4.14, H-6b‴) with C-9‴′ (*δ*_C_ 168.6). Therefore, the structure of **1** was determined as shown (Figures 3, 4).

**TABLE 2:** ^1^H NMR (400 MHz) and^13^C NMR (100 MHz) data of compound **1** in CD_3_OD-DCl (9:1).

Position	1	
	*δ*_H_ (*J* in Hz)	*δ*_C_ type
1	—	—
2	—	164.0, C
3	—	145.0, C
4	8.92 s	139.1, CH
5	—	158.8, C
6	6.57 d (1.28)	103.5, CH
7	—	174.2, C
8	6.65 d (1.28)	95.5, CH
9	—	157.3, C
10	—	113.1, C
1′	—	119.8, C
2′	7.68 d (2.6)	112.6, CH
3′	—	147.1, C
4′	—	143.9, C
5′	—	147.1, C
6′	7.68 d (2.6)	112.6, CH
3-*O*-Glc (Glc A)		
1″	5.34 d (7.5)	103.5, CH
2″	3.99 dd (8.8, 7.5)	84.1, CH
3″	3.80 dd (9.0, 9.0)	77.1, CH
4″	3.56 m	70.3, CH
5″	3.50 m	78.2, CH
6″	3.70 dd (12.2, 5.1), 3.84 dd (12.2, 1.9)	61.9, CH_2_
2″-*O*-Glc (Glc B)		
1‴	4.80 d (7.6)	106.0, CH
2‴	3.38 m	75.9, CH
3‴	3.48 m	77.1, CH
4‴	3.33 m	71.1, CH
5‴	3.42 m	75.2, CH
6‴	4.00 dd (12.1, 5.5), 4.14 dd (12.1, 2.0)	64.1 CH_2_
*p-coumaric acid*		
1‴′	—	126.5, C
2‴′	7.07 d (8.6)	130.9, CH
3‴′	6.72 d (8.6)	116.8, CH
4‴′	—	160.8, C
5‴′	6.72 d (8.6)	116.8, CH
6‴′	7.07 d (8.6)	130.9, CH
7‴′	7.09 d (16.0)	146.3, CH
8‴′	5.71 d (16.0)	113.1, CH
9‴′	—	168.6, C

3′-hydroxylikarisoside C (**2**, peak **16**) was obtained as yellow powder, HRESIMS data (*m/z* 841.2773 [M+H]^+^, calcd for C_38_H_49_O_21_^+^ 841.2766) gave a molecular formula of C_38_H_48_O_21_. The 1H NMR data (Table 3) displayed characteristic signals of an isopentane group at *δ*_H_ 1.61 (3H, s), 1.71 (3H, s), 5.18 (1H, m), 3.42 (1H, m), and 3.58 (1H, m). The presence of three sugar moieties was deduced from anomeric hydrogen signals at 4.99 (1H, d, *J* = 7.6 Hz), *δ*_H_ 4.24 (1H, d, *J* = 7.9 Hz), and 5.50 (1H, d, *J* = 1.6 Hz), which was further confirmed to be d-glucose and l-rhamnose from acid hydrolysis. The large coupling constant observed with the anomeric hydrogens (*J* = 7.9 and 7.6 Hz) indicated a *β*-configuration for glucose moieties, whereas the *α*-configuration of rhamnose was deduced from the chemical shifts of C-3‴ (*δ*_C_ 70.2) and C-5‴ (*δ*_C_ 70.5) (Agrawal, 1992) (Table 3). The NMR data of two were highly similar to those of ikarisoside C, except for the existence of a 1,3,4-trisubstuted benzene ring, deduced from the ABX coupling system at *δ*_H_ 7.32 (1H, dd, *J* = 8.3, 2.2 Hz), 6.89 (1H, d, *J* = 8.3 Hz), and 7.41 (1H, d, *J* = 2.2 Hz) in **2**, rather than an AABB coupling system observed for ikarisoside C, revealing the presence of a hydroxyl group at C-3′ in **2**. HMBC correlations of H-1‴ (*δ*_H_ 5.50) and H-1″ (*δ*_H_ 4.99) with C-3 (*δ*_C_ 134.5) and C-7 (*δ*_C_ 160.4), respectively, supported that the 8-prenylated quercetin was glycosylated with Rha at C-3 and Glc A at C-7. The Rha (2→1) Glc B linkage was confirmed with the HMBC correlation from H-1‴′ (*δ*_H_ 4.24) of Glc B to C-2‴ (*δ*_C_ 81.5) of Rha. Therefore, the structure of **2** was identified as shown (Figures 3, 4).

**TABLE 3:** ^1^H NMR (500 MHz) and^13^C NMR (125 MHz) data of compounds **2–4** in DMSO-*d*_6_.

Position	2		3		4	
	*δ*_H_ (*J* in Hz)	*δ*_C_ type	*δ*_H_ (*J* in Hz)	*δ*_C_ type	*δ*_H_ (*J* in Hz)	*δ*_C_ type
1	—	—	—	―	—	—
2	—	157.5, C	—	157.5, C	—	157.8, C
3	—	134.5, C	—	134.2, C	—	134.1, C
4	—	178.3, C	—	178.3, C	—	178.1, C
5	—	159.01, C	—	159.1, C	—	159.1, C
6	6.60 s	98.0, CH	6.60 s	98.0, CH	6.61 s	98.0, CH
7	—	160.4, C	—	160.4, C	—	160.5, C
8	—	108.2, C	—	108.2, C	—	108.2, C
9	—	152.3, C	—	152.8, C	—	152.9, C
10	—	105.4, C	—	105.3, C	—	105.4, C
11	3.42, 3.58 m	21.4, CH_2_	3.42, 3.58 m	21.4, CH_2_	3.43, 3.56 m	21.5, CH_2_
12	5.18 m	122.2, CH	5.18 m	122.2, CH	5.15 m	122.1, CH
13	—	131.2, C		131.2, C	—	131.2, C
14	1.61 s	25.5, CH_3_	1.61 s	25.5, CH_3_	1.61 s	25.5, CH_3_
15	1.71 s	17.4, CH_3_	1.71 s	17.5, CH_3_	1.71 s	17.5, CH_3_
1′	―	120.4, C	―	120.11, C	―	120.5, C
2′	7.41 d (2.2)	115.6, CH	7.41 d (2.2)	115.5, CH	7.36 d (2.2)	115.8, CH
3′	—	145.5, C	—	145.5, C	—	145.5, C
4′	—	149.3, C	—	149.5, C	—	149.0, C
5′	6.89 d (8.3)	115.6, CH	6.89 d (8.4)	115.6, CH	6.87 d (8.4)	115.5, CH
6′	7.32 dd (8.3, 2.2)	121.0, CH	7.32 dd (8.4, 2.2)	120.9, CH	7.31 dd (8.4, 2.2)	121.2, CH
7-*O*-Glc						
1″	4.99 d (7.6)	100.6, CH	4.98 d (6.7)	100.6, CH	4.99 d (7.7)	100.6, CH
2″	3.31 m	73.4, CH	3.29 m	73.4, CH	3.30 m	73.4, CH
3″	3.29 m	76.6, CH	3.31 m	76.6, CH	3.34 m	76.6, CH
4″	3.17 m	69.7, CH	3.15 m	69.7, CH	3.17 m	69.7, CH
5″	3.42 m	77.2, CH	3.42 m	77.2, CH	3.42 m	77.2, CH
6″	3.46 m, 3.71 dd (12.0, 1.9)	60.6, CH_2_	3.47 m, 3.71 dd (11.9, 1.9)	60.6, CH_2_	3.48 m, 3.71 dd (12.0, 1.9)	60.6, CH_2_
3-*O*-Rha						
1‴	5.50 d (1.6)	101.0, CH	5.32 d (1.4)	100.9, CH	5.39 d (1.6)	100.5, CH
2‴	4.15 dd (4.2, 1.6)	81.5, CH	4.07 dd (3.7, 1.4)	80.1, CH	4.12 dd (3.3, 1.6)	75.7, CH
3‴	3.61 m	70.2, CH	3.60 m	70.3, CH	3.66 m	70.3, CH
4‴	3.14 m	71.8, CH	3.12 m	71.7, CH	3.14 m	72.0, CH
5‴	3.58 m	70.5, CH	3.66 m	70.4, CH	3.26 m	70.7, CH
6‴	0.89 d (6.2)	17.9, CH_3_	0.90 d (6.2)	17.9, CH_3_	0.84 d (6.2)	17.9, CH_3_
2‴-*O*-Glc or 2‴-*O*-Xyl or 2‴-*O*-Rha						
1‴′	4.24 d (7.9)	106.3, CH	4.16 d (7.7)	106.5, CH	4.88 (1.7)	101.7, CH
2‴′	2.97 m	73.9, CH	2.94 m	73.8, CH	3.68 m	70.1, CH
3‴′	3.14 m	69.0, CH	3.05 m	76.2, CH	3.34 m	70.5, CH
4‴′	3.15 m	76.2, CH	3.17 m	69.3, CH	3.16 m	72.0, CH
5‴′	2.95 m	76.5, CH	3.45 m	65.7, CH_2_	3.37 m	68.8, CH
6‴′	3.31 m, 3.46 m	60.2, CH_2_	2.90 m		1.09 d (6.2)	17.6, CH_3_

3′-hydroxylepimedoside E (**3**, peak **17**) was isolated as yellow powder, HRESIMS data (*m/z* 811.2677 [M+H]^+^, calcd for C_37_H_47_O_20_^+^ 811.2661) gave a molecular formula of C_37_H_46_O_20._ The NMR data were similar to those of epimedoside E, except for an ABX coupling system at *δ*_H_ 7.32 (1H, dd, *J* = 8.4, 2.2 Hz), 6.89 (1H, d, *J* = 8.4 Hz) and 7.41 (1H, d, *J* = 2.2 Hz) in **3**, instead of a AABB coupling system in epimedoside E, supporting the existence of a hydroxyl group at C-3′ in **3**. The three sugar moieties were confirmed to be d-glucose, l-rhamnose and d-xylose from acid hydrolysis. The large coupling constant observed with the anomeric hydrogen (*J* = 6.7 Hz) indicated a *β*-configuration for the glucose moiety, whereas the *α*-configuration for rhamnose and *β*-configuration for xylose was deduced from chemical shifts of C-3‴ (*δ*_C_ 70.3) and C-5‴ (*δ*_C_ 70.4) for rhamnose, and C-3‴′ (*δ*_C_ 76.2) and C-5‴′ (*δ*_C_ 65.7) for xylose, respectively (Agrawal, 1992) (Table 3). HMBC correlations of H-1‴ (*δ*_H_ 5.32) and H-1″ (*δ*_H_ 4.98) with C-3 (*δ*_C_ 134.2) and C-7 (*δ*_C_ 160.4), respectively, supported that the 8-prenylated quercetin was glycosylated with Rha at C-3 and Glc at C-7. The Rha (2→1) Xyl linkage was confirmed with the HMBC correlation from H-1‴′ (*δ*_H_ 4.16) of Xyl to C-2‴ (*δ*_C_ 80.1) of Rha. Therefore, the structure of **3** was elucidated as shown (Figures 3, 4).

3′-hydroxyldiphylloside B (**4**, peak **18**) was obtained as yellow powder, HRESIMS data (*m/z* 825.2832 [M+H]^+^, calcd for C_38_H_49_O_20_^+^ 825.2817) gave a molecular formula of C_38_H_48_O_20_. The NMR data were similar to those of diphylloside B, except for an ABX coupling system at *δ*_H_ 7.31 (1H, dd, *J* = 8.4, 2.2 Hz), 6.87 (1H, d, *J* = 8.4 Hz) and 7.36 (1H, d, *J* = 2.2 Hz) in **4**, instead of a AABB coupling system in diphylloside B, supporting the existence of a hydroxyl group at C-3′ in **4**. The presence of three sugar moieties was deduced from anomeric hydrogen signals at *δ*_H_ 4.99 (1H, d, *J* = 7.7 Hz), 4.88 (1H, d, *J* = 1.7 Hz), and 5.39 (1H, d, *J* = 1.6 Hz), which was further confirmed to be d-glucose and l-rhamnose from acid hydrolysis. The large coupling constant observed with the anomeric hydrogen (*J* = 7.7 Hz) indicated a *β*-configuration for glucose moiety, whereas the *α*-configuration of rhamnose moieties was deduced from the chemical shifts of C-3‴ (*δ*_C_ 70.3) and C-5‴ (*δ*_C_ 70.7) of Rha A, and C-3‴′ (*δ*_C_ 70.5) and C-5‴′ (*δ*_C_ 68.8) of Rha B (Agrawal, 1992) (Table 3). HMBC correlations of H-1‴ (*δ*_H_ 5.39) and H-1″ (*δ*_H_ 4.99) with C-3 (*δ*_C_ 134.1) and C-7 (*δ*_C_ 160.5), respectively, supported that the 8-prenylated quercetin was glycosylated with Rha A at C-3 and Glc at C-7. The Rha A (2→1) Rha B linkage was confirmed from the HMBC correlation from H-1‴′ (*δ*_H_ 4.88) of Rha B to C-2‴ (*δ*_C_ 75.7) of Rha A. Therefore, the structure of **4** was identified as shown (Figures 3, 4).

### Effects of different *Epimedium* flavonols on testosterone production in rat leydig cells

**FIGURE 5:**
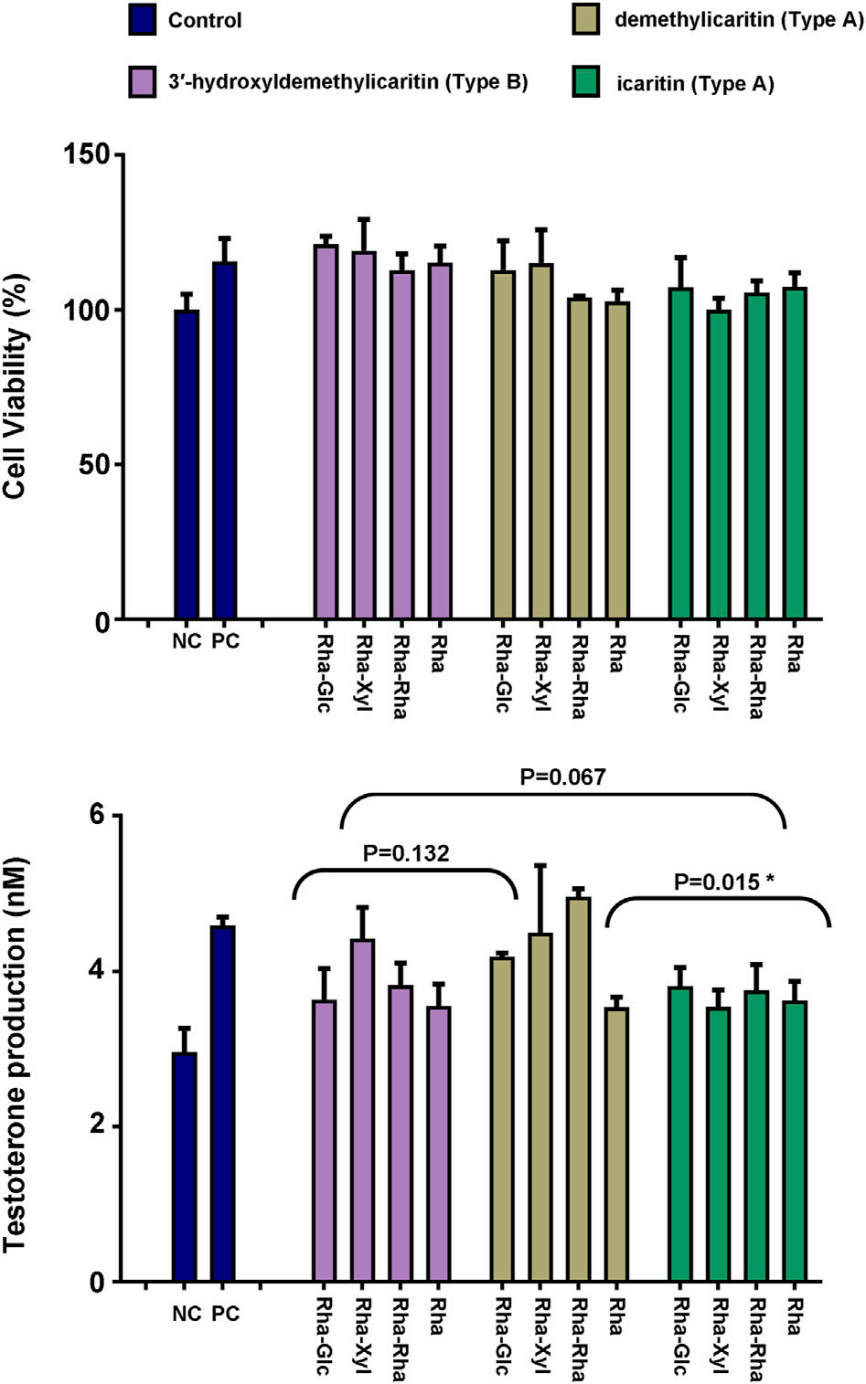
Effect of 12 different key *Epimedium* flavonols on testosterone production in rat Leydig cells. Cell viability was analyzed by MTT assay. Results were depicted as mean +/− SD, *p < 0.05.

*Epimedium* flavonols was known to enhance the sexual abilities, and it has been widely reported that testosterone plays an important modulatory role in the control of sexual activities. Therefore, the new compounds **1**–**4** and other nine structurally related *Epimedium* flavonols were evaluated for their testosterone production-promoting activities in rat primary Leydig cells with forskolin as a positive control (Table 4), and the corresponding peaks in the LC-MS analysis of endogenous extract were labelled in Table 4. Cell viability was analyzed through MTT assay at a dosage of 5 μmol L^−1^, 1 μmol L^−1^ and 0.2 μmol L^−1^, and at the treatment conditions, the culture cells was all more than 90% of viability (Figure 5). At a dosage of 5 μmol L^−1^, all the tested compounds significantly increased the testosterone production over the untreated condition (1.12−1.56 fold) (Figure 5). The testosterone production-promoting effects of different types of *Epimedium* flavonols was in the order: subtype demethylicaritin in Type A (1.29−1.56 fold) > subtype 3′-hydroxyldemethylicaritin in type B (1.15−1.31 fold), including the compounds **2**−**4** > subtype icaritin in Type A (1.12−1.18 fold). Significant difference (*p* = 0.015) was found between subtype demethylicaritin in Type A and subtype icaritin in Type A, but was not detected between subtype demethylicaritin in Type A and subtype 3′-hydroxyldemethylicaritin in Type B (*p* = 0.132), and between subtype 3′-hydroxyldemethylicaritin in Type B and subtype icaritin in Type A (*p* = 0.067), see Figure 5.

**TABLE 4:** List of compounds used in cellular viability and testosterone assays.

Compound number (isolated)	Compound name	Type of *Epimedium* flavonols	Modification on ring B	Glycosylation on C-3 of ring C	Glycosylation on C-7 of ring a
**2**	3′-hydroxylikarisoside C (new)	Type B: 3′-hydroxyl-demethylicaritin	3′-OH, 4′-OH	Rha (2–1) Glc	Glc
**3**	3′-hydroxylepimedoside E (new)			Rha (2–1) Xyl	Glc
**4**	3′-hydroxyldiphylloside B (new)			Rha (2–1) Rha	Glc
**5**	3′-hydroxylepimedoside A			Rha	Glc
**7**	ikarisoside C		4′-OH	Rha (2–1) Glc	Glc
**8**	epimedoside E			Rha (2–1) Xyl	Glc
**9**	diphylloside B			Rha (2–1) Rha	Glc
**10**	epimedoside A	Type A: demethylicaritin		Rha	Glc
References standard	epimedin A		4′-OCH_3_	Rha (2–1) Glc	Glc
References standard	epimedin B			Rha (2–1) Xyl	Glc
References standard	epimedin C	Type A: icaritin		Rha (2–1) Rha	Glc
References standard	icariin			Rha	Glc
**1**	delphinidin-3-*O*-*p*-coumaroyl-sophoroside (new)	—			

## Discussion

### Classification and distribution pattern of *Epimedium* flavonols

In our LC-MS analyses conducted separately on petals and inner sepals from flowers of *E. acuminatum*, a total of 32 compounds were identified, including 22 flavonols, six anthocyanins, three phenolic acids and one alkaloid. Among the 22 flavonols, 12 were found to be Type A *Epimedium* flavonols, four were identified as Type B *Epimedium* flavonols, and the remaining six were identified as non-prenylated flavonols. For Type A *Epimedium* flavonols, both of the subtypes (icaritin and demethylicaritin) were detected, whereas only flavonols of 3′-hydroxyldemethylicaritin subtype was observed for Type B. The petals and inner sepals of *E. acuminatum* were highly similar in chemical constituents, except that petunidin derivates and delphinidin-3,5-di-*O*-glucoside were only present in the inner sepals. The content of *Epimedium* flavonols in the petals was higher than that in the inner sepals. The LC-MS profiling led to the isolation of one new anthocyanin (**1**), three new 8-prenylated quercetin glycosides (Type B) (**2**−**4**), and six known flavonols (**5**−**10**) from the flowers of *E. acuminatum*.

The new 8-prenylated quercetin glycosides (**2**−**4**), in combination with the previous discovered 3′-hydroxylepimedoside A (Li et al., 2017), exhibited common patterns of glycosylation, indicating that 8-prenylated quercetin glycosides should also be a key form of *Epimedium* flavonols, which has not been previously elucidated. Therefore, we proposed that the key classification of 8-prenylated flavonols in *Epimedium* plants (Figure 1) should be based on the initial backbone of kampferol (Type A) or quercetin (Type B). In *Epimedium* plants, Type A *Epimedium* flavonols were distributed more extensively than Type B. Subsequently, Type A and Type B *Epimedium* flavonols could be further divided into two subtypes based on the presence or absence of methylation on C-4′ of ring B (Figure 1). Clearly, the new 8-prenylated quercetin glycosides (**2**−**4**) isolated in our study completed the *Epimedium* flavonols of subtype 3′-hydroxyldemethylicaritin in Type B, and *Epimedium* flavonols of subtype 3′-hydroxylicaritin in Type B still remains incomplete. Besides, for plant flavonoids, it is common for methylation to occur on the C-3′ position of ring B (eg. isorhamnetin, syringetin, laricitrin, petunidin) (Koirala et al., 2016). However, for *Epimedium* flavonols, it seems a lot easier for methylation to occur on the C-4′ position rather than the C-3′ position of ring B, suggesting the uniqueness of *O*-methyltransferases in *Epimedium* plants. Additionally, previous studies had deduced dihydroicaritin derivates in *Epimedium* species (Yu et al., 2016; Zhou et al., 2021) from LC-MS data, but their fragmentation patterns and retention time were identical with the new 8-prenylated quercetin glycosides isolated in this study, suggesting that the dihydroicaritin derivates reported in previous LC-MS studies of *Epimedium* should be corrected to the 3′-hydroxyldemethylicaritin derivates reported here. This correction could further be correlated with our literature survey, in which dihydroicaritin derivates has never been isolated from *Epimedium* species.

**FIGURE 6:**
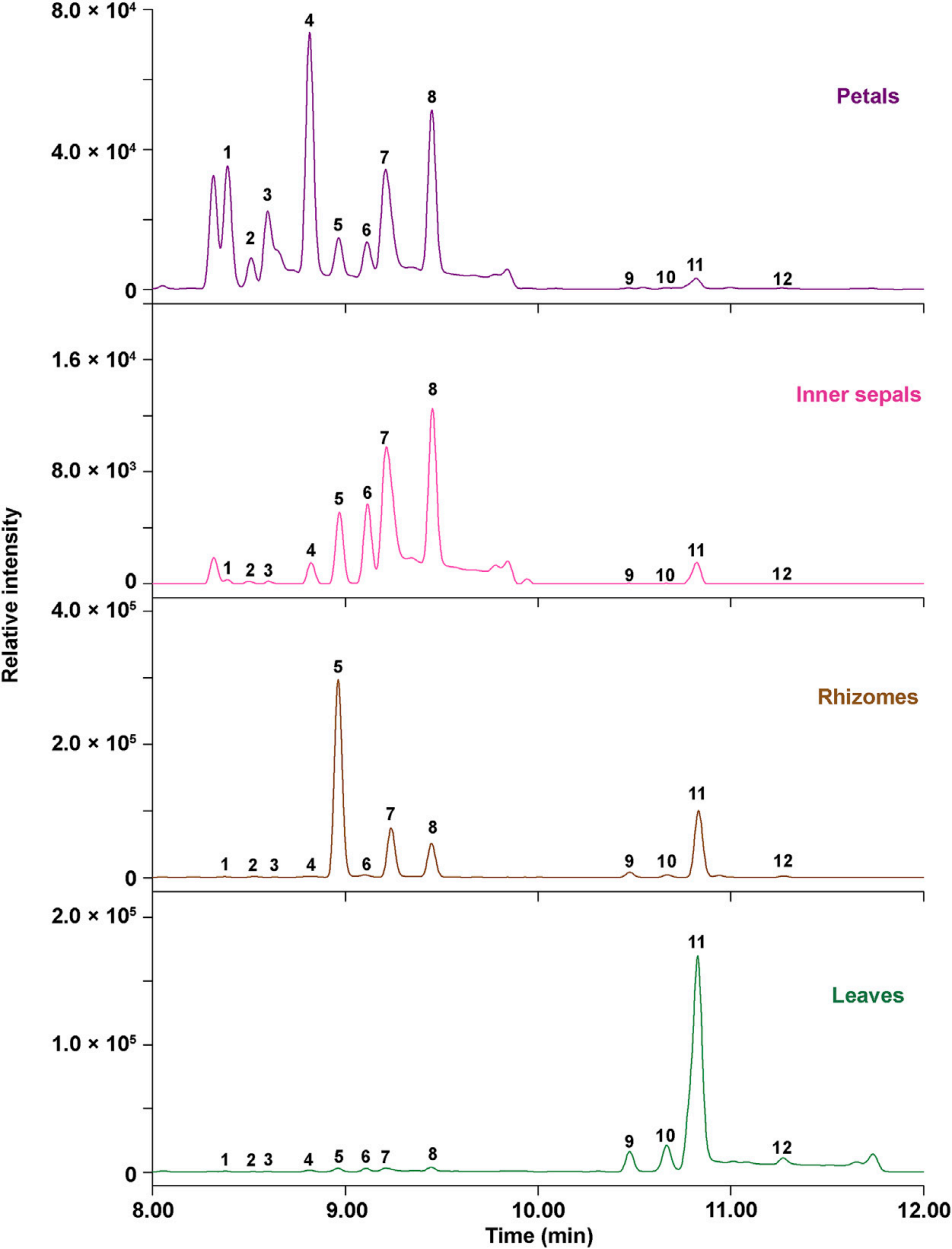
Ultra-high-performance liquid (UPLC) chromatograms at 360 nm for relative quantification of 12 key *Epimedium* flavonols in different tissues of *E. acuminatum*.

The relative content of the 12 key *Epimedium* flavonols was measured and found to be different in petals, inner sepals, rhizomes, and leaves of *E. acuminatum* (Figure 6; Table 5). In general, the relative content of Type A *Epimedium* flavonols was higher than Type B. The relative content of *Epimedium* flavonols of subtype icaritin in Type A (4′-methoxyl) in different tissues was in the order: Leaves > rhizomes >> petals > inner sepals, whereas the highest content of subtype demethylicaritin in Type A (4′-hydroxyl) was found in rhizomes, followed by petals, inner sepals, and then leaves. *Epimedium* flavonols of subtype 3′-hydroxyldemethylicaritin in Type B (4′-hydroxyl) was highest in petals, and only trace amount was detected in inner sepals, rhizomes, and leaves, which should be reason why this subtype of *Epimedium* flavonols has remained elusive for long. On the whole, the different accumulation pattern of hydroxylated and methylated *Epimedium* flavonols in different tissues should indicate different biological functions in the corresponding tissues, which are worthy to be investigated. According to studies on biological functions of hydroxylated and methylated flavonoids in plants, hydroxylated flavonoids might contribute to the formation of colors and tastes of flowers which could facilitates pollination, whereas methylation improves chemical stability and membrane permeability, and maybe also the toxicity of flavonoids, which might help to resist microorganism and herbivores (Lahtinen et al., 2004; Alseekh et al., 2020).

**TABLE 5:** Relative quantification of 12 key *Epimedium* flavonols in the MeOH (containing 0.1% acetic acid) extract of petals, inner sepals, rhizomes, and leaves from *E. acuminatum*.

Peak no.	Rt	Compound name	Type of *Epimedium* flavonols	Modification on ring B	Glycosylation on C-3 of ring C	Glycosylation on C-7 of ring A	Inner sepals (μg/g)	Petals (μg/g)	Rhizomes (μg/g)	Leaves (μg/g)
1	8.39	3′-hydroxylikarisoside C		3′-OH, 4′-OH	Rha (2–1) Glc	Glc	7.835	173.497	21.107	14.179
2	8.51	3′-hydroxylepimedoside E	Type B: 3′-hydroxyl-demethylicaritin		Rha (2–1) Xyl	Glc	7.162	232.298	0.000	6.451
3	8.65	3′-hydroxyldiphylloside B			Rha (2–1) Rha	Glc	0.000	113.307	5.944	0.000
4	8.83	3′-hydroxylepimedoside A			Rha	Glc	37.911	1,247.303	6.342	18.653
5	8.96	ikarisoside C		4′-OH	Rha (2–1) Glc	Glc	243.678	344.893	2,245.239	98.789
6	9.11	epimedoside E			Rha (2–1) Xyl	Glc	216.981	351.314	41.229	51.791
7	9.25	diphylloside B	Type A: demethylicaritin		Rha (2–1) Rha	Glc	205.579	354.095	1,461.200	65.928
8	9.46	epimedoside A			Rha	Glc	720.794	1,480.260	1,380.037	147.282
9	10.49	epimedin A		4′-OCH_3_	Rha (2–1) Glc	Glc	26.734	33.153	516.994	985.179
10	10.66	epimedin B			Rha (2–1) Xyl	Glc	35.459	43.141	336.901	1,267.573
11	10.83	epimedin C	Type A: icaritin		Rha (2–1) Rha	Glc	254.065	331.808	3,362.020	4,102.047
12	11.27	icariin			Rha	Glc	20.136	39.542	253.649	568.042

### Different types of *Epimedium* flavonols exhibit different testosterone production-promoting activities

To investigate the SAR of different types of *Epimedium* flavonols on testosterone production-promoting activities, the new compounds (**1**−**4**) and nine structurally related compounds were tested, which could be divided into three major types of *Epimedium* flavonols based on their chemical structures except for the new compound **1** (Table 4). As a result, all the tested *Epimedium* flavonols and the new compound 1 were found to increase testosterone production over the control. Furthermore, most of the compounds did not significantly affect the cell proliferation except for the new compound **1**, whose impact on the increase of testosterone production may be partly due to the enhancement of cell proliferation. Based on the bioactivities and chemical structures of tested compounds, it can be concluded that either methylation at C-4′ position or hydroxylation at C-3′ position of ring B could decrease the biological activity (Figure 5; Table 4). In addition, the comparison among compounds within each subtypes suggested a trend that the addition of sugar moieties to the C-3 position of ring C would increase the biological activity, which needs to be further investigated.

## Conclusion

Phytochemical investigation on petals and inner sepals from flowers of *E. acuminatum* was first carried out with LC-MS analysis, leading to identification of 32 compounds. Subsequently, the LC-MS profiling guided the discovery of three new 8-prenylated quercetin glycosides, one new anthocyanin and six known compounds. In combination with previous phytochemical studies on *Epimedium* plants, we proposed *Epimedium* flavonols to be classified into Type A (8-prenylated kaempferol based), including icaritin and demethylicaritin subtypes, and Type B (8-prenylated quercetin based), including 3′-hydroxylicaritin and 3′-hydroxyldemethylicaritin subtypes. The SAR study was conducted by comparing testosterone production-promoting activities of new compounds with nine related *Epimedium* flavonols, suggesting that either methylation at C-4′ position or hydroxylation at C-3′ position of ring B could decrease the biological activity.

## Methods and material

### Plant materials

Plants were grown in the Germplasm Repository (Guizhou province, China) at latitude 32.06 and longitude 131.31. Fresh petals, inner sepals, leaves, and rhizomes of *E. acuminatum* were collected, and quick-frozen in liquid nitrogen in the spring of 2021. The samples were later freeze-dried and stored at −20°C until extraction. Voucher specimens were deposited at the Herbarium of the Institute of Medicinal Plant Development, Beijing, China (IMPLAD), and were identified by Professor Baolin Guo (IMPLAD).

### General experimental procedures

High-resolution mass spectrometry was conducted on a Waters Acquity UPLC I-Class Plus-Xevo G2 XS Q-ToF (Waters Corporation, Milford, United States), equipped with an ESI source. The separation was carried out with a RP-C18 column (150 mm × 3.0 mm) with particle size of 1.8 µm (Agilent Technologies, Santa Clara, United States). The NMR spectra were measured on a Bruker Avance III 400 MHz and a 500 MHz spectrometer (Bruker, Rheinstetten, Germany), referenced to the solvent signals of CD_3_OD and DMSO-*d*_6_. Semipreparative HPLC was conducted on a Lumtech K-501 equipped with a K-2501 UV detector. A 250 mm × 50 mm, 10 μm, ODS column and a 250 mm × 10 mm, 5 μm, ODS-A column (YMC, Kyoto, Japan) were applied for obtaining desired fractions and further purification, respectively. Macroporous adsorption resin D101 (Meilun biotechnology, Dalian, China), and Sephadex LH-20 (40−70 μm) (Pharmacia Biotech AB, Uppsala, Sweden) were used for column chromatography. Chromatographic grade methanol and acetonitrile (Thermo Fisher Scientific, Waltham, United States) were used for preparative HPLC. Analytical grade methanol and acetic acid (Tianjin SaiFuRui Technology, Tianjin, China) was used for extraction and column chromatography. Reference standards including delphinindin-3,5-di-*O*-glucoside, delphinidin-3-*O*-glucoside, petunidin-3-*O*-glucoside, neochlorogenic acid, chlorogenic acid, and astragalin were purchased from Sigma-Aldrich Co., Ltd. (Burlington, United States), and reference standards of hyperoside, isoquercitrin, astragalin, magnoline, epimedins A−C, icariin and 2″-*O*-rhamnosylicariside II were purchased from Chengdu PUSH-biotechnology Co., Ltd. (Chengdu, China). Other reference standards used in this study were isolated and their structures were confirmed through a comparison of NMR and MS data with the literature.

### LC-ESI/Q-TOF/MS analyses

The samples of petals, inner sepals, leaves and rhizomes from *E. acuminatum*, separately, were extracted with MeOH (containing 0.1% acetic acid) at 4°C and were centrifuged for 10 min at 10,000 rpm. The supernatants were collected, dried with vaccum centrifuge concentrator (CV100-DNA, Aijimu, Beijing, China), and stored at −20°C until analysis. The dried extracts were re-dissolved in MeOH right before analysis and were examined on a liquid chromatography quadrupole time-of-flight mass spectrometer, Q-TOF-MS (Waters Xevo G2-XS QTOF) (Waters Corporation, Milford, United States) with an ESI source consisting of ACQUITY UPLC I-Class instrument (Waters Corporation, Milford, United States). The separation was carried out with a RP-C18 column (150 mm × 3.0 mm, 1.8 µm) at 40°C. The elution program consisted 0.1% formic acid (A) and acetonitrile (B) as the mobile phases, and a gradient elution profile was applied (flow rate of 0.4 ml min^−1^): 0−1 min (5% B), 1−8 min (5%–30% B), 8−12 min (30%–40% B), 12−16 min (40%–95% B), 16−17 min (95%–100% B), 17−21 min (100% B), 21−22 min (100% B−5% B), 22−25 min (5% B). The mass spectrometer operated in positive ion mode. ESI source parameters were as follows: capillary voltages 0.5 kV; desolvation gas flow 1000 L Hr^−1^ (N_2_). MS spectra were obtained over the range of *m/z* 100−1,200.

The compounds in sample extracts were identified by comparing with the retention times, characteristics of UV-Vis spectra of peaks and the mass spectrometric information of reference standards using software MassLynx v4.1. The relative content of 12 key *Epimedium* flavonols was calculated from peak areas of samples based on the intensity of the corresponding standard compounds.

### Extraction and isolation

A mixture of dried petals and inner sepals of *E. acuminatum* (100 g) were extracted in ultrasonic bath (1 h) for three times with 80% MeOH (containing 1% acetic acid) at room temperature in darkness. The mixture was filtered through a Buchner funnel and the pooled filtrates were concentrated under reduced pressure at 45°C. The extract was then partitioned against petroleum ether, and concentrated to obtain the crude anthocyanin-flavonol containing MeOH-aqueous extract.

The crude extract (82.0 g) was firstly passed through a macroporous adsorption resin D101 (un-polarity) with distilled water to remove most of the polysaccharides, and then the solvent was changed to 50% EtOH to elute anthocyanins and desired flavonols. The 50% EtOH residue (5.93 g) was first separated by semipreparative HPLC with a 250 mm × 50 mm, 10 μm, ODS column using solvents CH_3_CN (A) and 0.5% acetic acid (B). The elution profile consisted of 15% A for 25 min, and 20% A for 30 min to obtain three desired fractions (Fr.1−3).

Fr.1 (182.5 mg) was further separated by passing through Sephadex LH-20 (eluted with 40% MeOH) to yield compound **1** (105.0 mg).

Fr.2 (176.6 mg) was first separated by passing through Sephadex LH-20 with MeOH to obtain three subfractions (Fr.2A−2C). Fr.2A (53.1 mg) was further purified by semipreparative HPLC with a 250 mm × 10 mm, 5 μm, ODS-A column (YMC, Kyoto, Japan) using solvents CH_3_CN (A) and 0.025% formic acid (B). The elution profile consisted of a linear gradient from 17% to 21% A in 20 min, isocratic elution for the next 30 min, followed by 100% A for 15 min at a flow rate of 2.0 ml/min to afford compound **2** (9.0 mg, *t*_R_ = 40.6 min), compound **3** (8.6 mg, *t*_R_ = 43.3 min) and compound **4** (8.0 mg, *t*_R_ = 46.6 min).

Fr.2B (61.3 mg) and 20.5 mg of Fr.2C (50.8 mg) was separated using the same elution profile as Fr.2A, yielding compound **5** (22.5 mg, *t*_R_ = 48.9 min) and **6** (8.0 mg, *t*_R_ = 38.2 min), respectively. 80.9 mg of Fr.3 (243.2 mg) was subjected to semipreparative HPLC with a 250 mm × 10 mm, 5 *μ*m, ODS-A column (YMC, Kyoto, Japan) using solvents [CH_3_CN: 0.025% formic acid, (23:77, v/v)] at 2.0 ml/min to provide compound **7** (6.2 mg, *t*_R_ = 35.4 min), **8** (6.3 mg, *t*_R_ = 38.1 min), **9** (5.6 mg, *t*_R_ = 41.6 min), and **10** (17.6 mg, *t*_R_ = 43.5 min).

Delphinidin-3-*O*-*p*-coumaroyl-sophoroside **(1)**: dark purplish red powder. HRESIMS *m/z* 773.1924 [M]^+^ (calcd for C_36_H_37_O_19_^+^ 773.1929). ^1^H and ^13^C NMR (CD_3_OD-DCl) data, see Table 2.

3′-hydroxylikarisoside C **(2)**: yellow powder. HRESIMS *m/z* 841.2773 [M+H]^+^ (calcd for C_38_H_49_O_21_ 841.2766). ^1^H and ^13^C NMR (DMSO-*d*_6_) data, see Table 3.

3′-hydroxylepimedoside E **(3)**: yellow powder. HRESIMS *m/z* 811.2742 [M+H]^+^ (calcd for C_38_H_49_O_21_ 811.2661). ^1^H and ^13^C NMR (DMSO-*d*_6_) data, see Table 3.

3′-hydroxyldiphylloside B **(4)**: yellow powder. HRESIMS *m/z* 825.2897 [M+H]^+^ (calcd for C_38_H_49_O_21_ 825.2817). ^1^H and ^13^C NMR (DMSO-*d*_6_) data, see Table 3.

Six known flavonols were characterized as 3′-hydroxyl-epimedoside A (**5**) (Li et al., 2017), hyperoside (**6**) (Liu et al., 2015), ikarisoside C (**7**) (Fukai & Nomura, 1988), epimedoside E (**8**) (Mizuno et al., 1991), diphylloside B (**9**) (Mizuno et al., 1988), and epimedoside A (**10**) (Xu, 1982) through the comparison of NMR and MS data with the literature.

### Acid hydrolysis

Standard sugars and compounds **1**–**4** (each 1.0 mg) were dissolved in 6 mol L^−1^ CF_3_COOH (1 ml) and heated at 90°C for 2 h and cooled to room temperature. The hydrolysate was extracted with CHCl_3_ for three times, and the aqueous layer was concentrated to obtain the residue containing sugars. The obtained residues were dissolved in pyridine (200 μl), and L-cysteine methyl ester hydrochloride (1 mg) was added and heated at 60°C for 1 h. Subsequently, o-tolyl isothiocyanate (10 μl) was added and heated at 60°C for another 1 h (Mitaine-Offer et al., 2010). After the reaction, the supernatants were filtrated and subjected to UPLC analysis (Vanquish Flex UHPLC system equipped with CAD detector) (Thermo Fisher Scientific, Waltham, United States) using a 100 mm × 2.1 mm, 1.8 μm, HSS T3 column (Waters Corporation, Milford, United States). The elution program consisted of a linear gradient of CH_3_CN in water (containing 0.1% formic acid, v/v) from 20% to 30% for 8 min (flow rate: 0.6 ml/min). The atomization temperature and wave filtering time were set at 35°C and 1 s, respectively. For compound **2** and **4**, derivatives of l-rhamnose and d-glucose were detected. However, for compound **3**, d-xylose was also observed. For compound **1**, only derivative of d-glucose was detected [*t*_R_ 4.43 min for d-glucose, *t*_R_ 6.31 min for l-rhamnose, and *t*_R_ 4.79 for d-xylose].

### Cellular viability and testosterone assays

The rat primary Leydig cells were prepared and cultured as previously described with modifications (Sharma et al., 2006; Chang et al., 2011). Briefly, these cells were initially plated at a density of 1 × 10^6^/ml with 2 ml culture medium in 6-well plates at the conditions: 37°C with 5% CO_2_. During the period of cell culture, the morphological changes of cells were monitored with an inverted microscope (Nikon Eclipse Ti2, Japan). To measure the purity of rat primary Leydig cells, 3*β*-HSD staining method was used (Chiao et al., 2002). For cellular viability assays, tested rat primary Leydig cells were plated at a density of 8,000 live cells per well in 96-well culture plates, which were placed in a 37°C, 5% CO_2_ incubator for 24 h before the activity assay. Before the assay, all the wells in culture plates were replaced with fresh 100 μl of culture medium, and the cells were treated with different tested compounds in a 37°C, 5% CO_2_ incubator for 72 h.

The cellular viability was evaluated using the MTT proliferation assay. In the MTT proliferation assay, the positive control samples were set with the treatment of forskolin (5 μmol L^−1^, 1 μmol L^−1^, and 0.2 μmol L^−1^), and the negative control samples were set without any compounds. For each experimental group, three replicates were set. During the assay, the diluted MTT solution was added to each well and incubated for 4 h. The supernatant was discarded, 100 μl of DMSO was added to each well, and the absorbance was measured at 570 nm by a microplate reader (Synergy HT, BioTek Instruments, Vermont, United States). Testosterone secreted into the culture medium was measured using testosterone ELISA kits according to the manufacturer’s instructions (Nanjing Jiancheng Biological Technology, Nanjing, China).

### Statistical analysis

Statistical analysis of the measurement of testosterone was performed *via* a two**-**tailed student’s *t*-test with two-sample equal variance.

## Data Availability

The original contributions presented in the study are included in the article/Supplementary Materials, further inquiries can be directed to the corresponding authors.
