# Supplementary material for: New 8-prenylated quercetin glycosides from the flowers of Epimedium acuminatum and their testosterone production-promoting activities
Source: Front Chem. 2022 Oct 10;10:1014110. doi: 10.3389/fchem.2022.1014110 (PMC9588921; doi:10.3389/fchem.2022.1014110)
Supplement: Supplementary file 1 [file DataSheet1.PDF]

Supplementary Material

Contents

1. **NMR spectra of compounds 1-4** 1
   - **Figure S1.** ^{1}H-NMR spectrum of compound **1** in CD_3OD-DCl (9:1) (400 MHz) 1
   - **Figure S2.** ^{13}C-NMR spectrum of compound **1** in CD_3OD-DCl (9:1) (100 MHz) 2
   - **Figure S3.** ^{1}H-^{1}H COSY spectrum of compound **1** 3
   - **Figure S4.** HSQC spectrum of compound **1** 4
   - **Figure S5.** HMBC spectrum of compound **1** 5
   - **Figure S6.** ^{1}H-NMR spectrum of compound **2** in DMSO-d_6 (500 MHz) 6
   - **Figure S7.** ^{13}C-NMR spectrum of compound **2** in DMSO-d_6 (125 MHz) 7
   - **Figure S8.** ^{1}H-^{1}H COSY spectrum of compound **2** 8
   - **Figure S9.** HSQC spectrum of compound **2** 9
   - **Figure S10.** HMBC spectrum of compound **2** 10
   - **Figure S11.** ^{1}H-NMR spectrum of compound **3** (500 MHz) 11
   - **Figure S12.** ^{13}C-NMR spectrum of compound **3** (125 MHz) 12
   - **Figure S13.** ^{1}H-^{1}H COSY spectrum of compound **3** 13
   - **Figure S14.** HSQC spectrum of compound **3** 14
   - **Figure S15.** HMBC spectrum of compound **3** 15
   - **Figure S16.** ^{1}H-NMR spectrum of compound **4** (500 MHz) 16
   - **Figure S17.** ^{13}C-NMR spectrum of compound **4** (125 MHz) 17
   - **Figure S18.** ^{1}H-^{1}H COSY spectrum of compound **4** 18
   - **Figure S19.** HSQC spectrum of compound **4** 19
   - **Figure S20.** HMBC spectrum of compound **4** 20
2. **Chemical structures of compounds 5-10** 1

1. NMR spectra of compounds 1-4

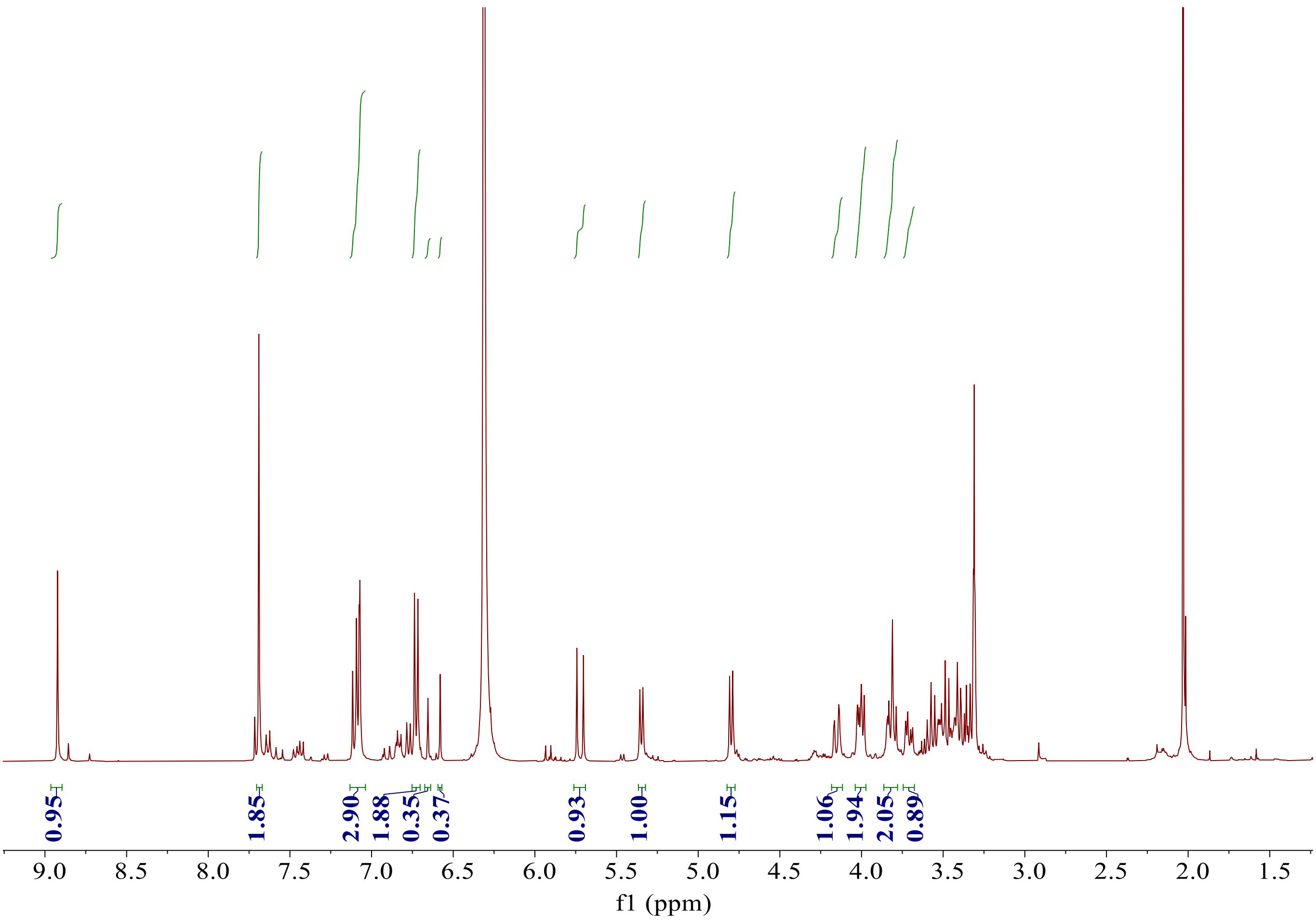

Figure S1. ^{1}H-NMR spectrum of compound **1** in CD_3OD-DCl (9:1) (400 MHz)

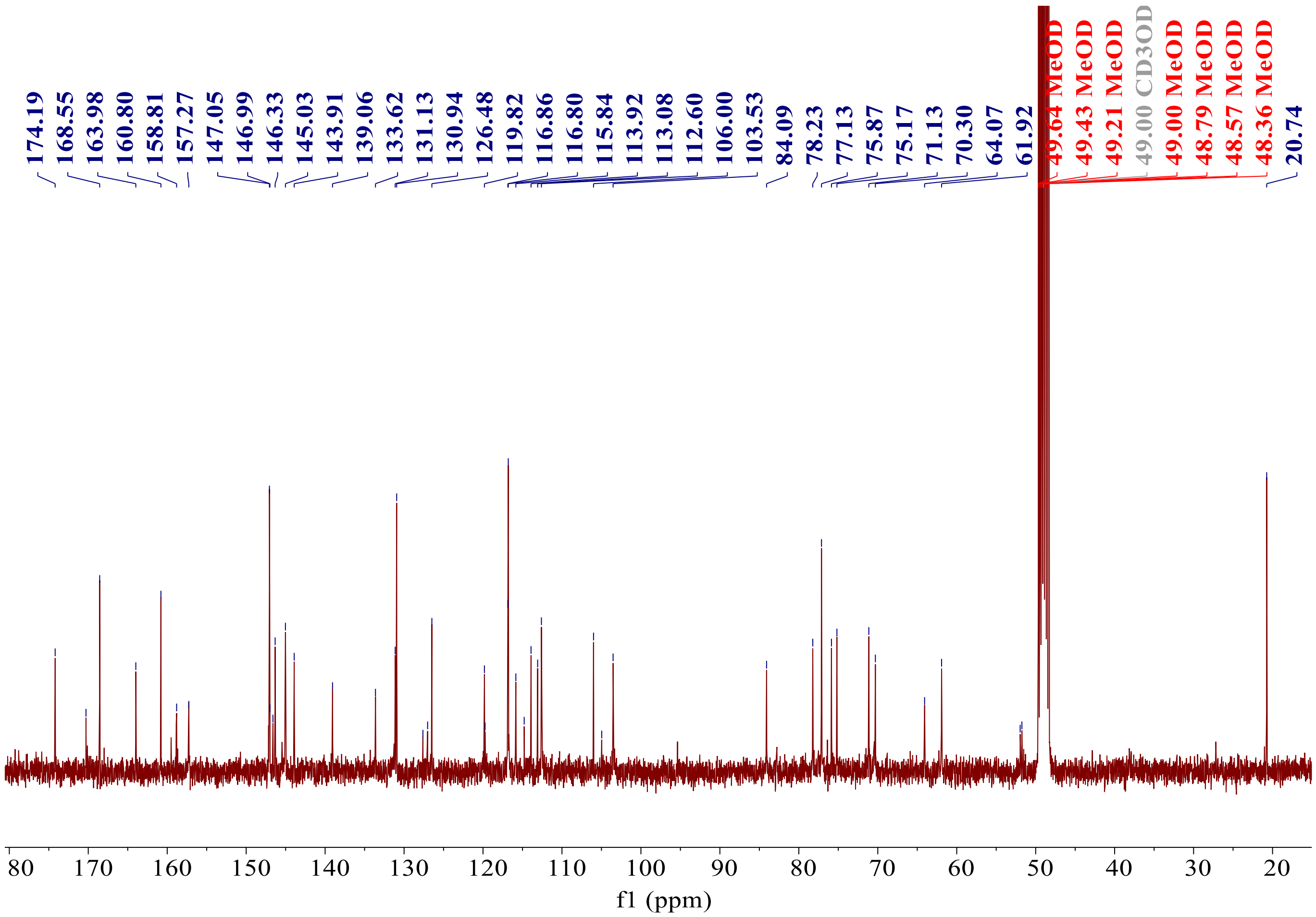

Figure S2. ^{13}C-NMR spectrum of compound **1** in CD_3OD-DCl (9:1) (100 MHz)

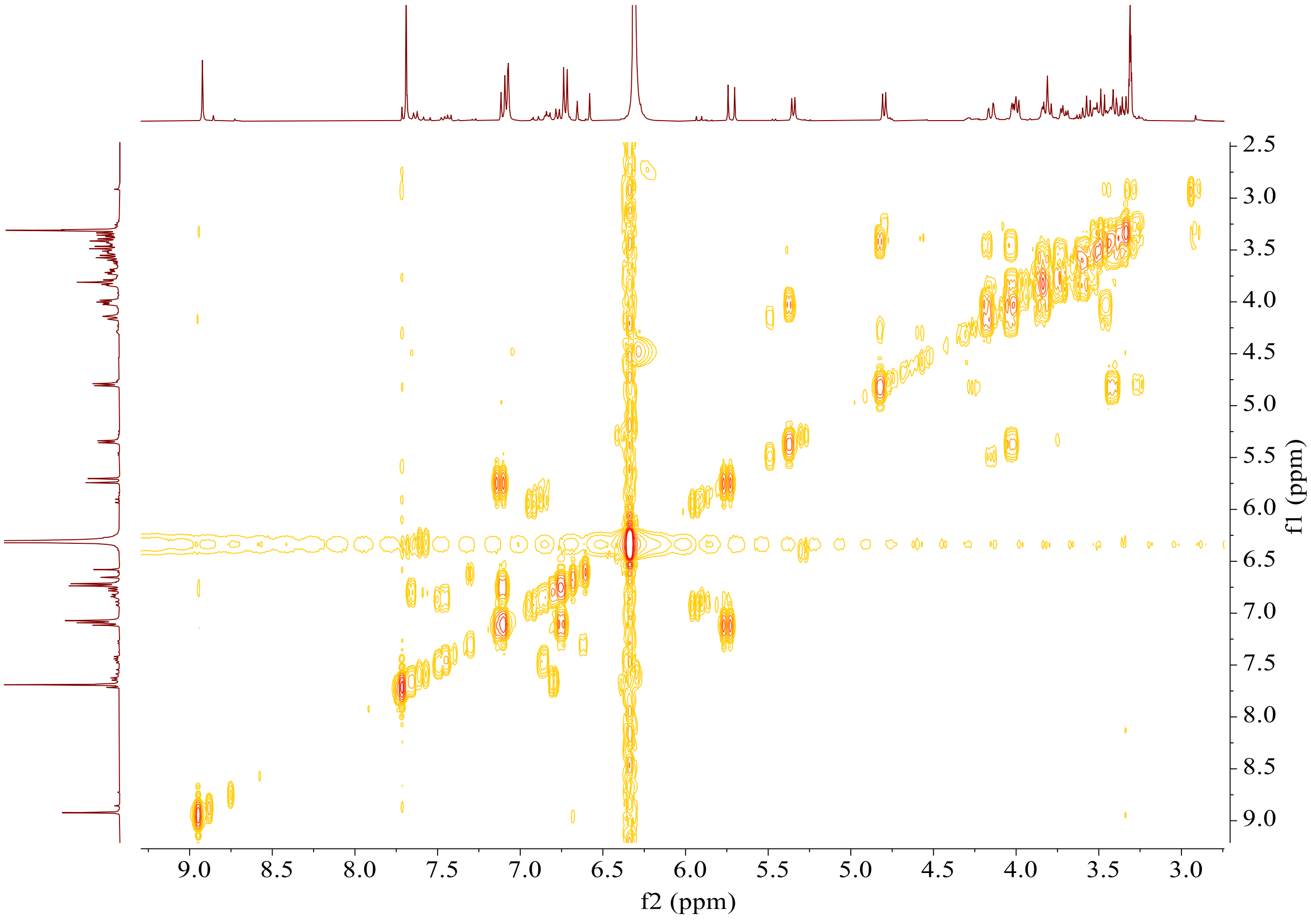

Figure S3. ^{1}H-^{1}H COSY spectrum of compound **1**

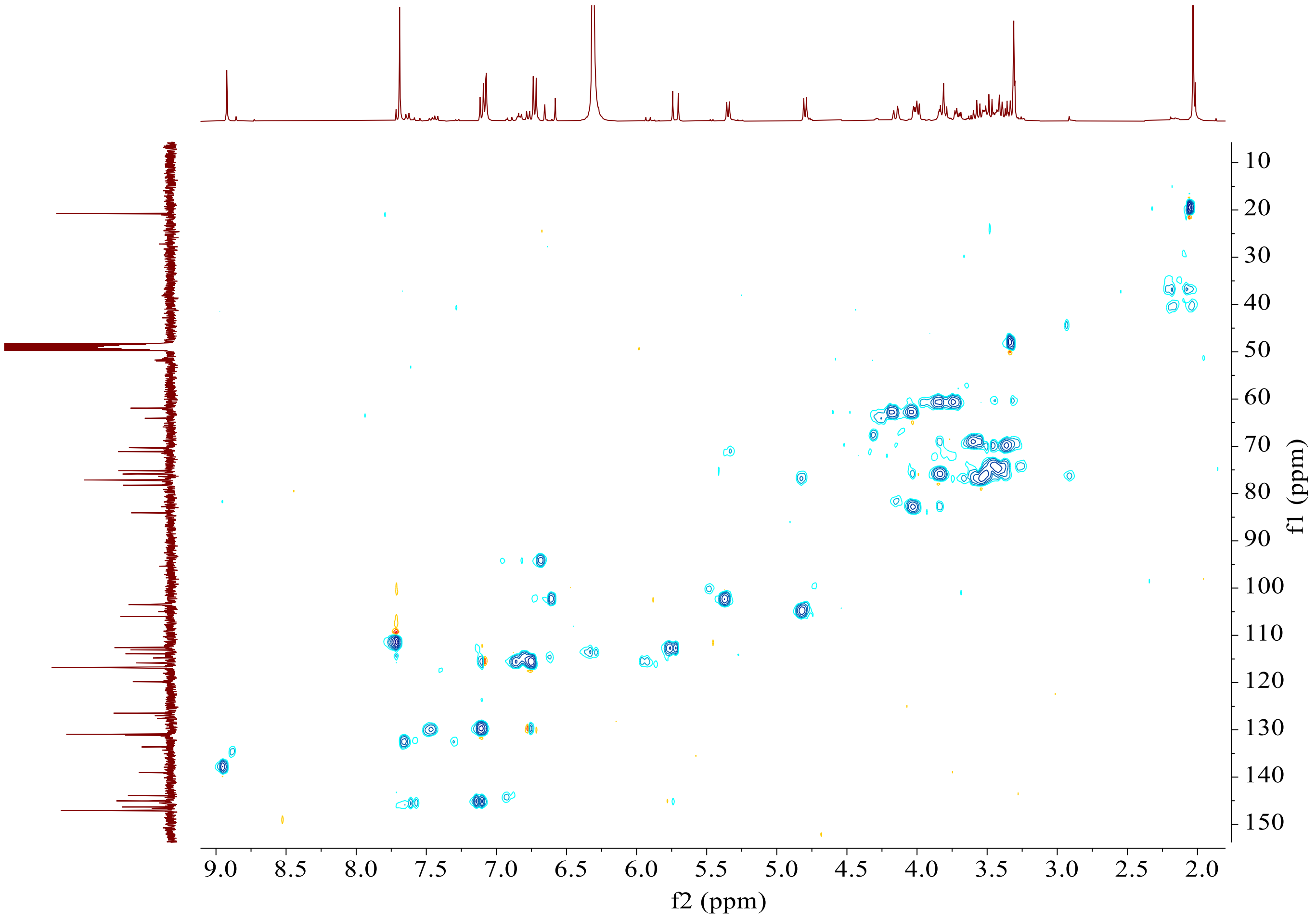

Figure S4. HSQC spectrum of compound **1**

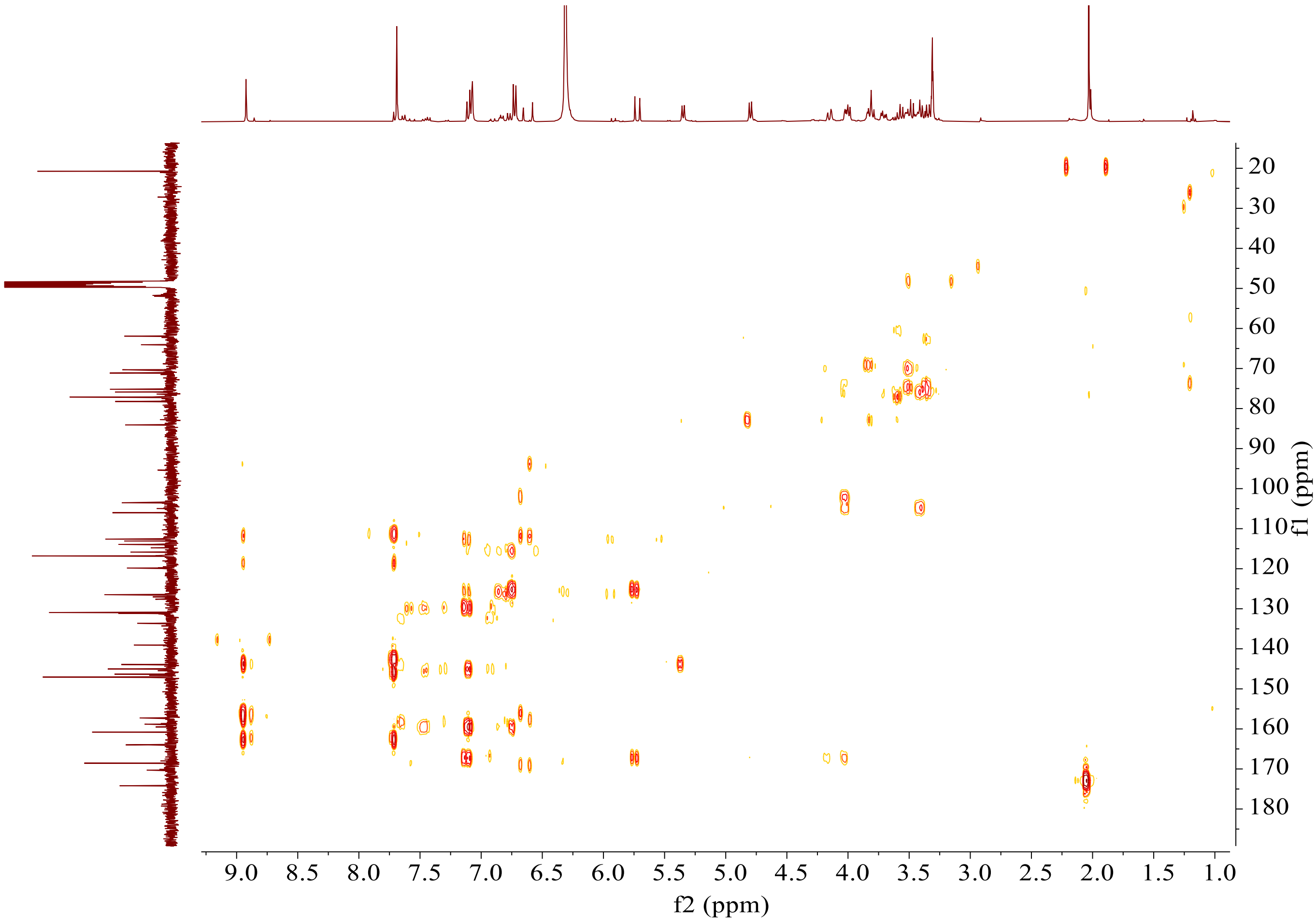

Figure S5. HMBC spectrum of compound **1**

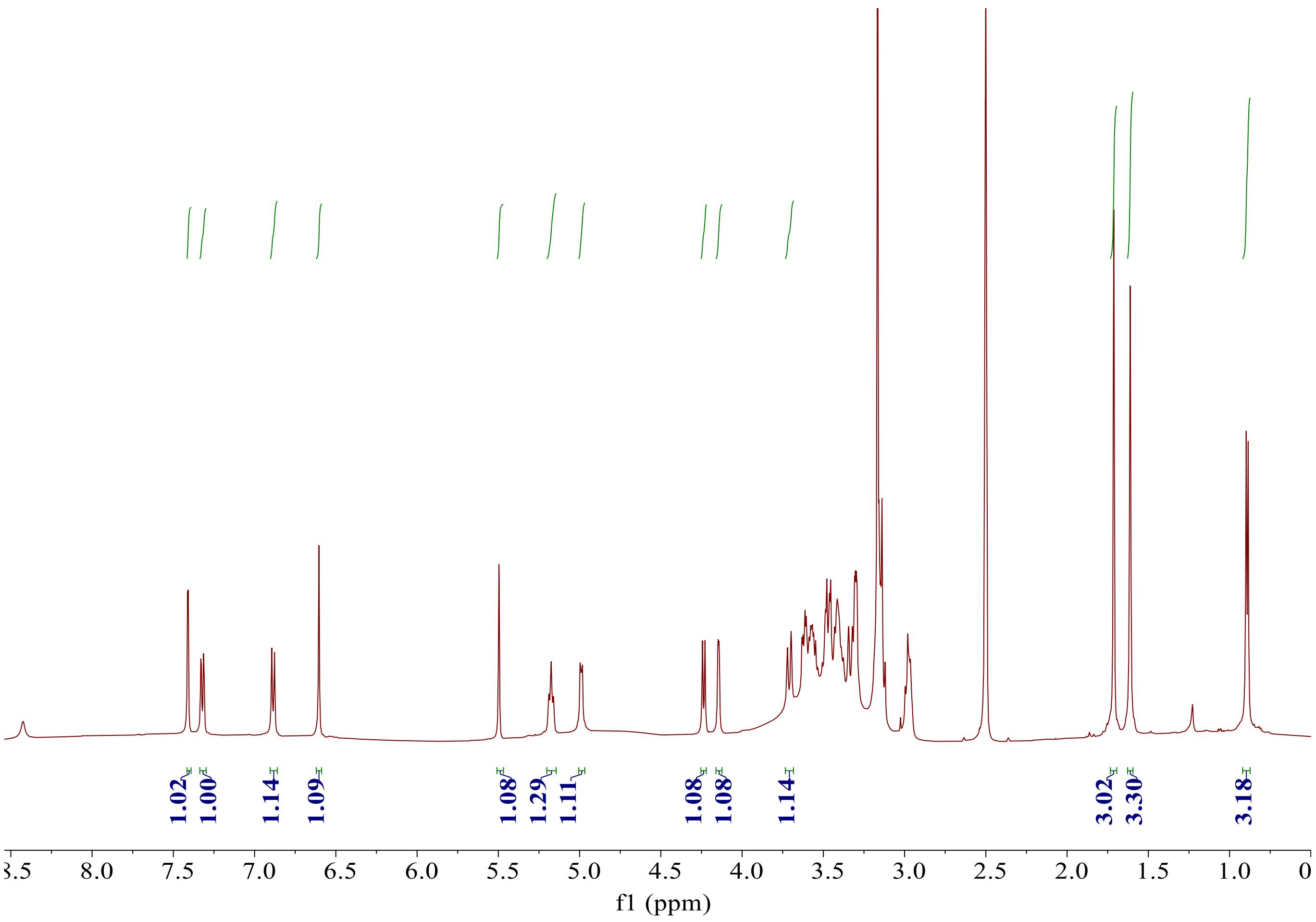

Figure S6. ^{1}H-NMR spectrum of compound **2** in DMSO-d_6 (500 MHz)

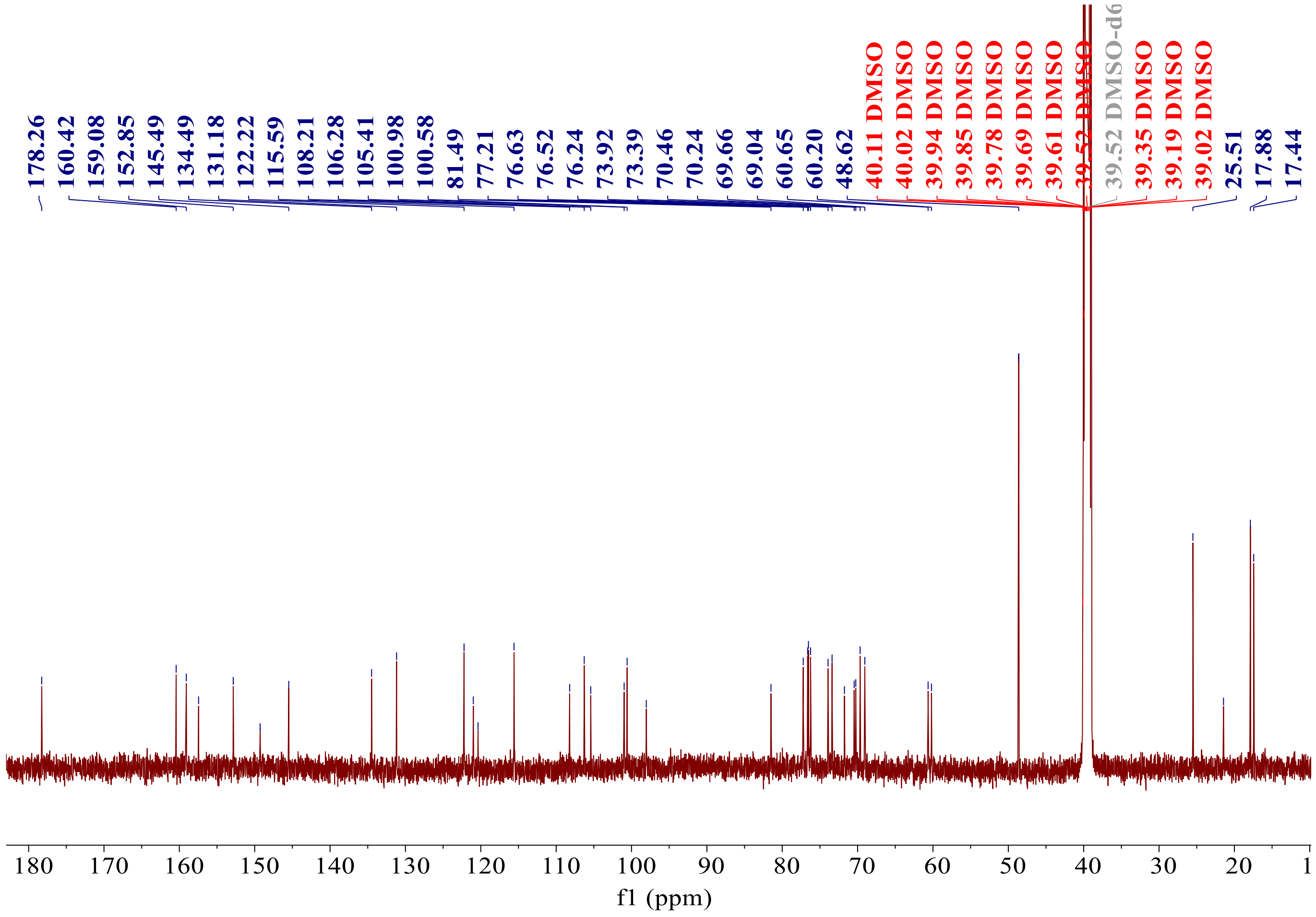

Figure S7. ^{13}C-NMR spectrum of compound **2** in DMSO-d_6 (125 MHz)

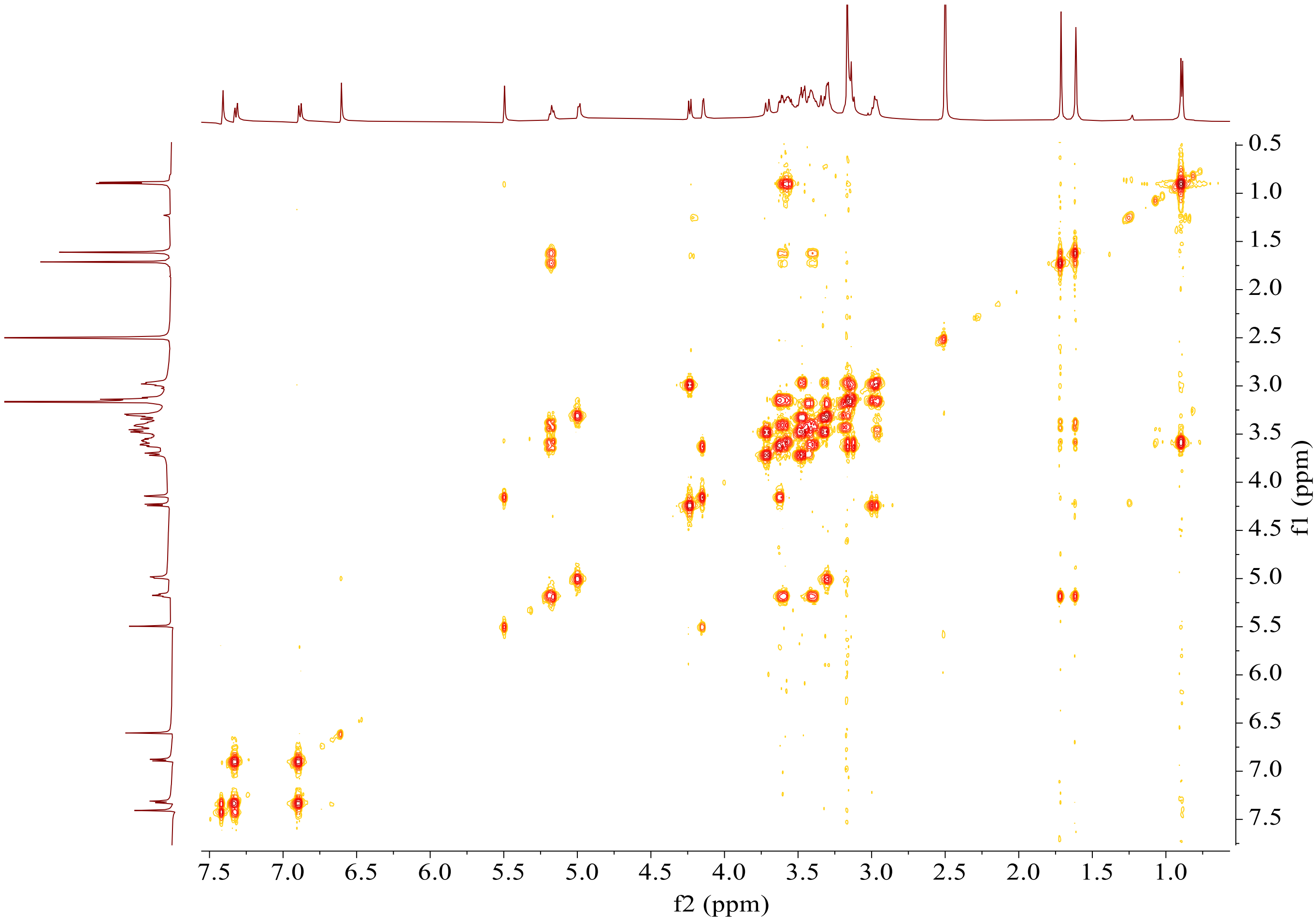

Figure S8. ^{1}H-^{1}H COSY spectrum of compound **2**

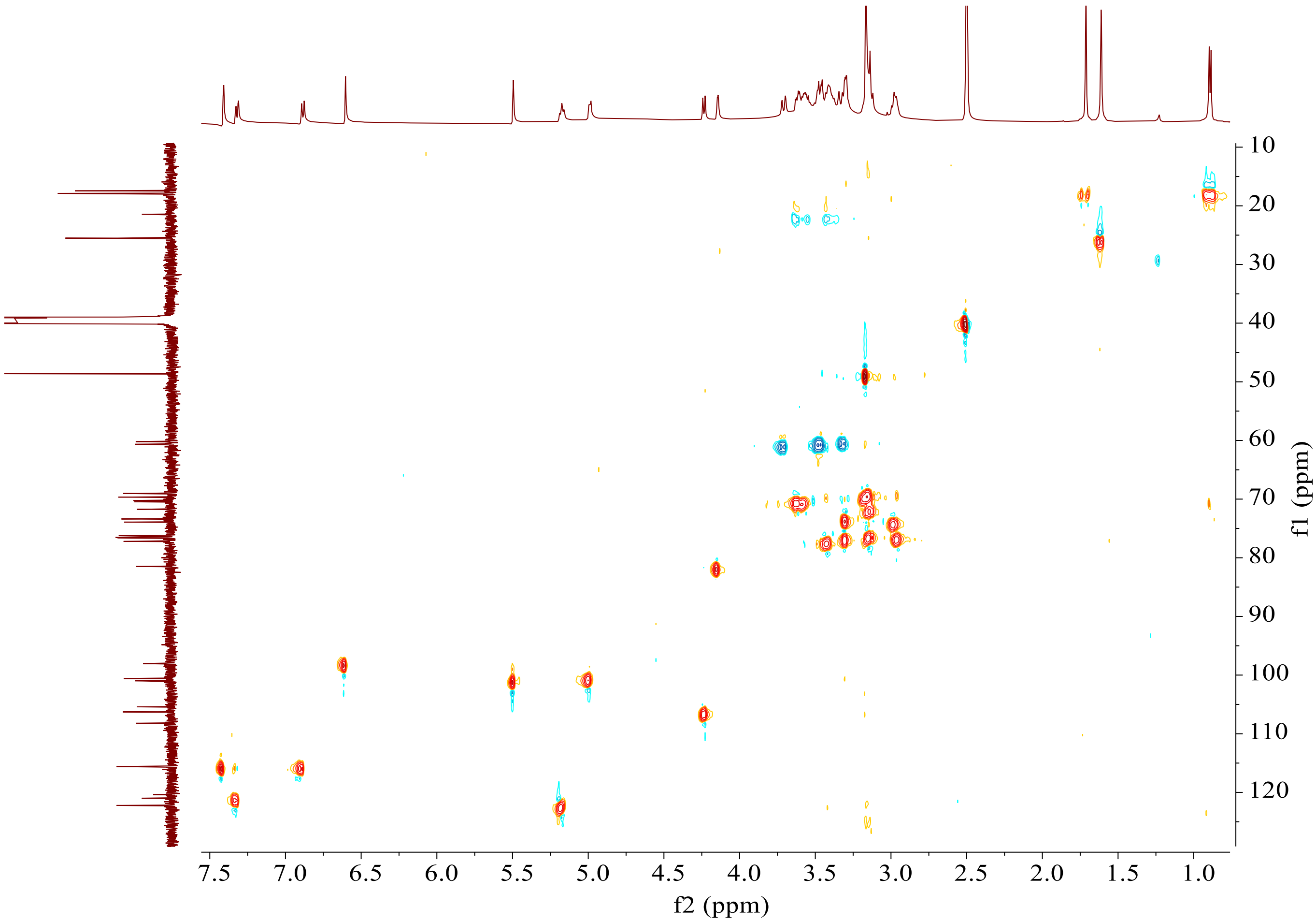

Figure S9. HSQC spectrum of compound **2**

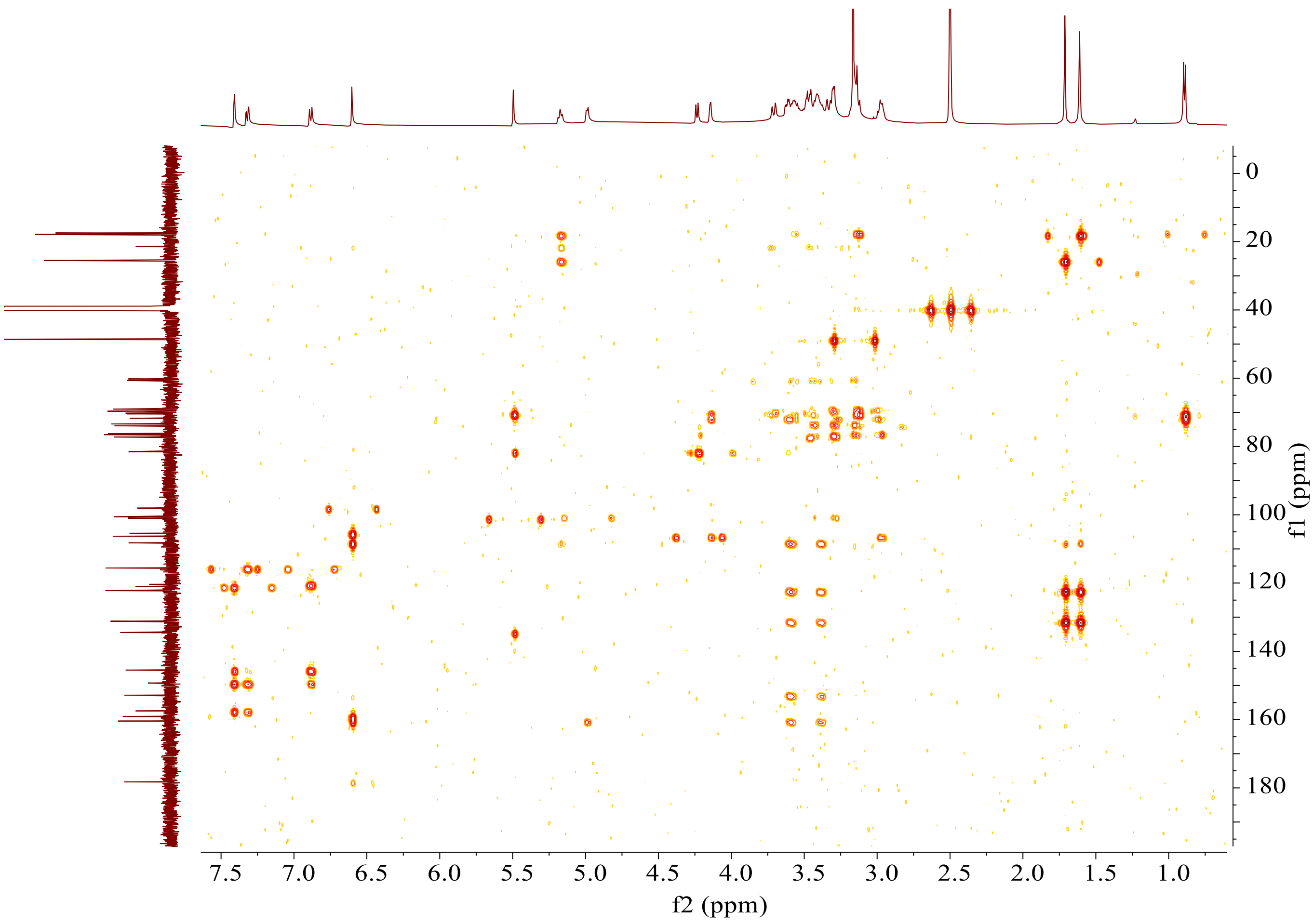

Figure S10. HMBC spectrum of compound **2**

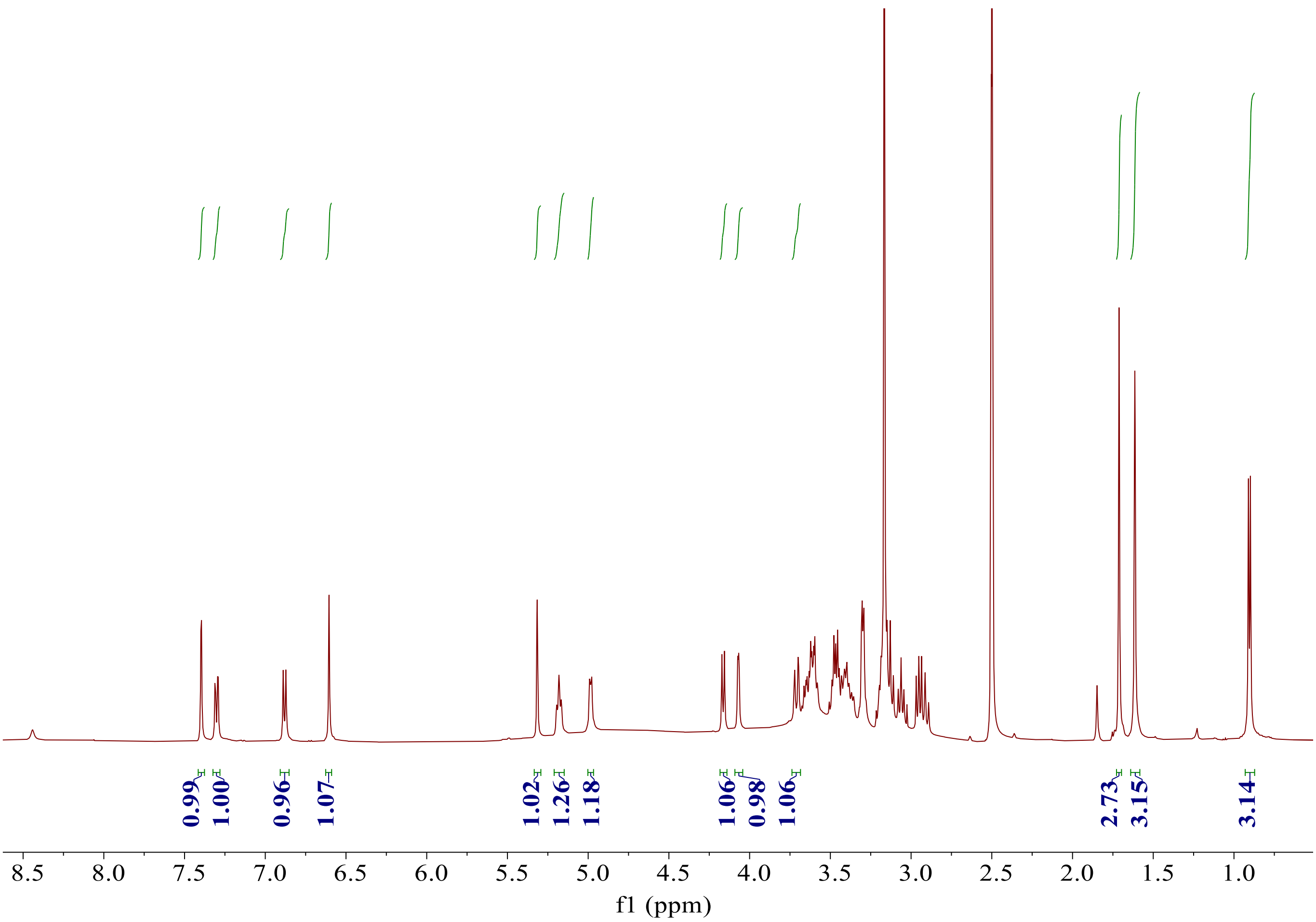

Figure S11. ^{1}H-NMR spectrum of compound **3** (500 MHz)

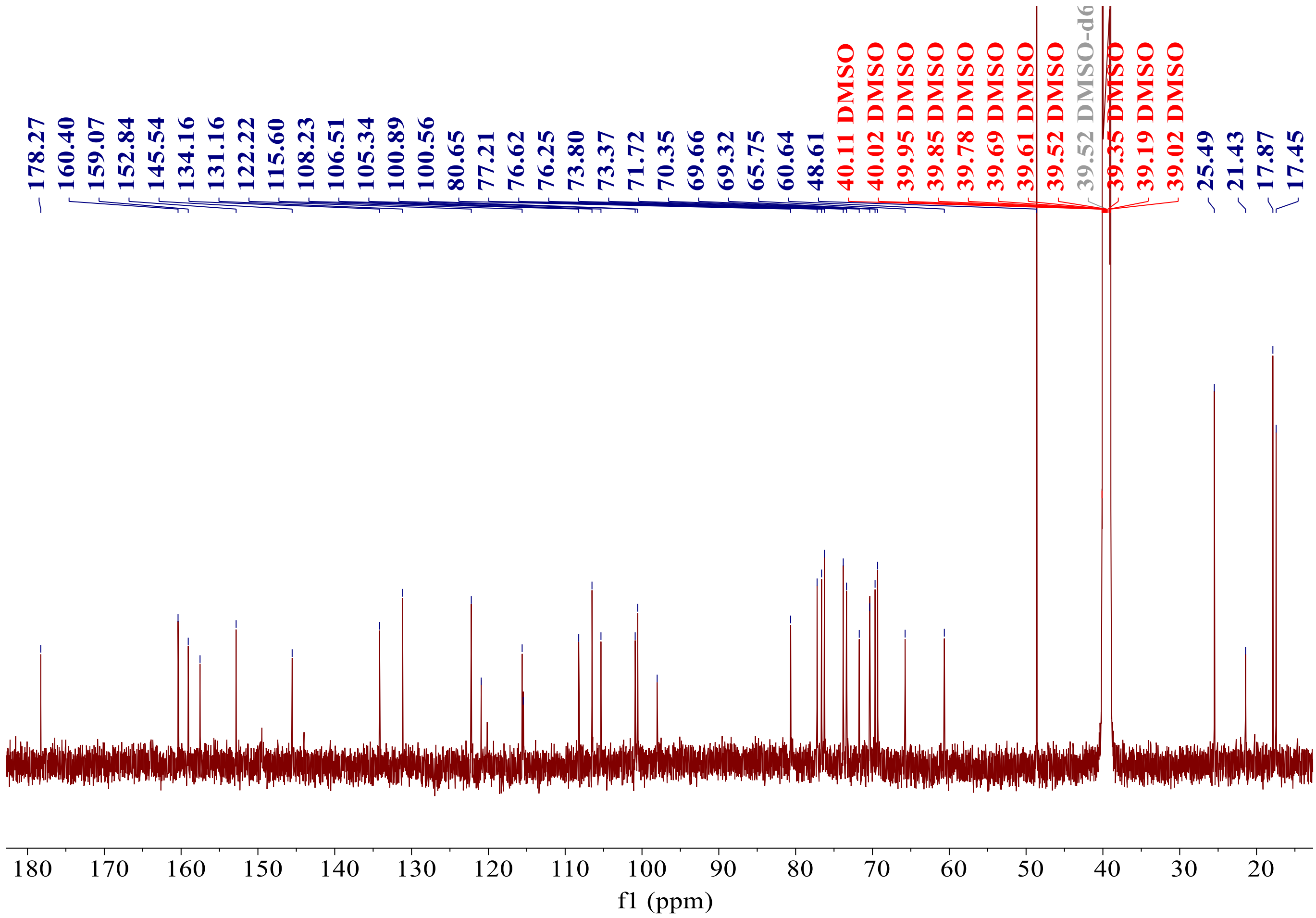

Figure S12. ^{13}C-NMR spectrum of compound **3** (125 MHz)

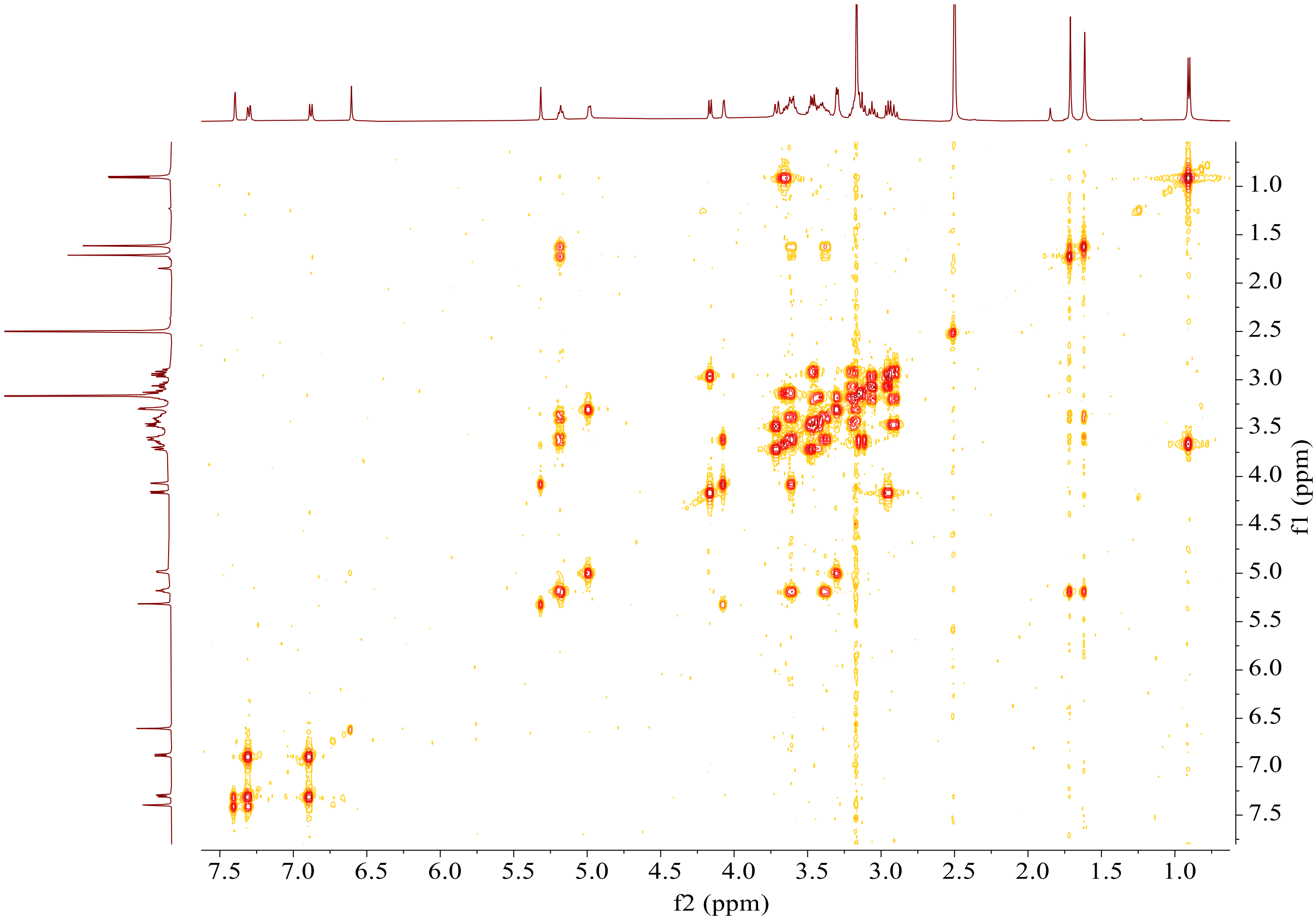

Figure S13. ^{1}H-^{1}H COSY spectrum of compound **3**

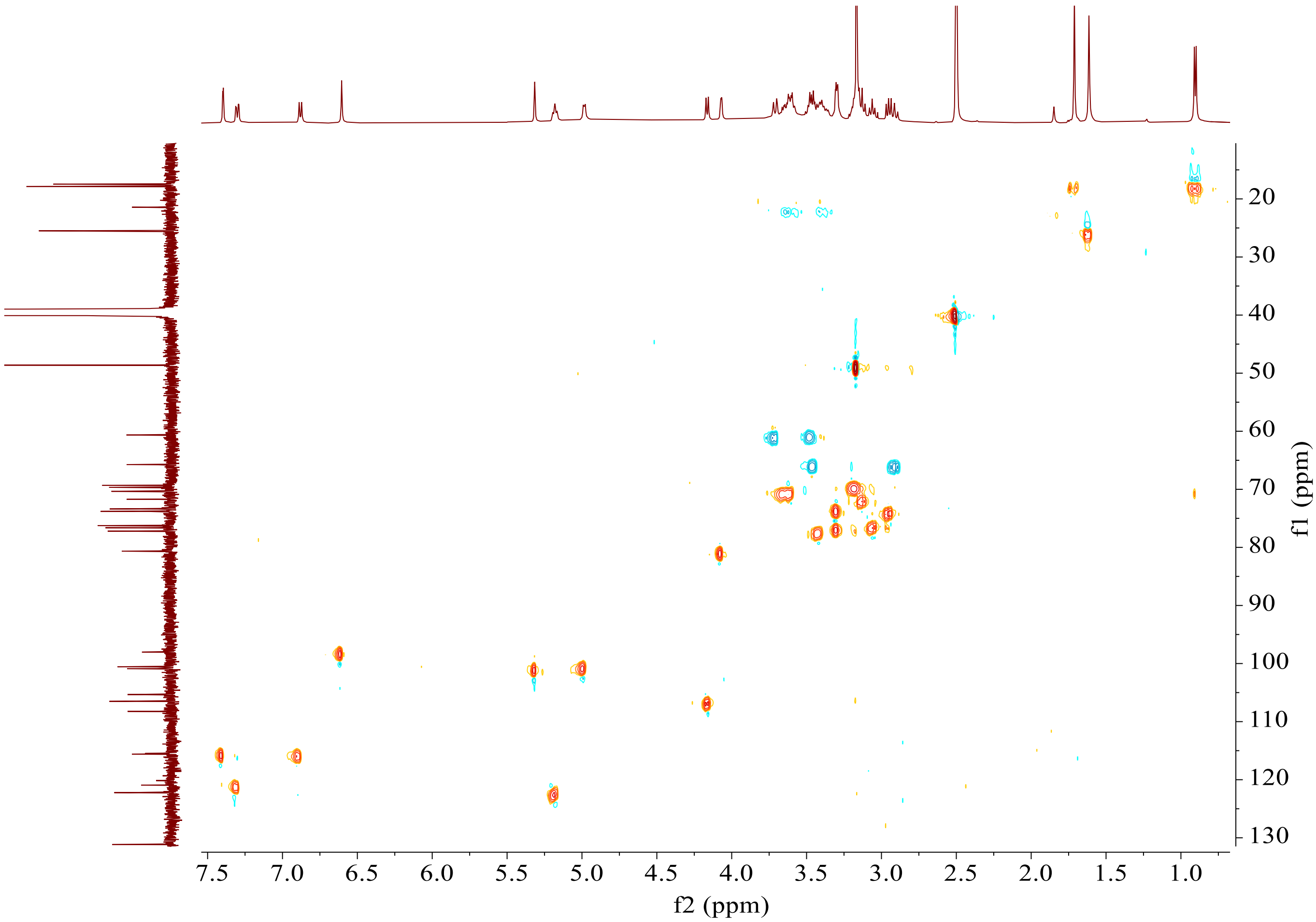

Figure S14. HSQC spectrum of compound **3**

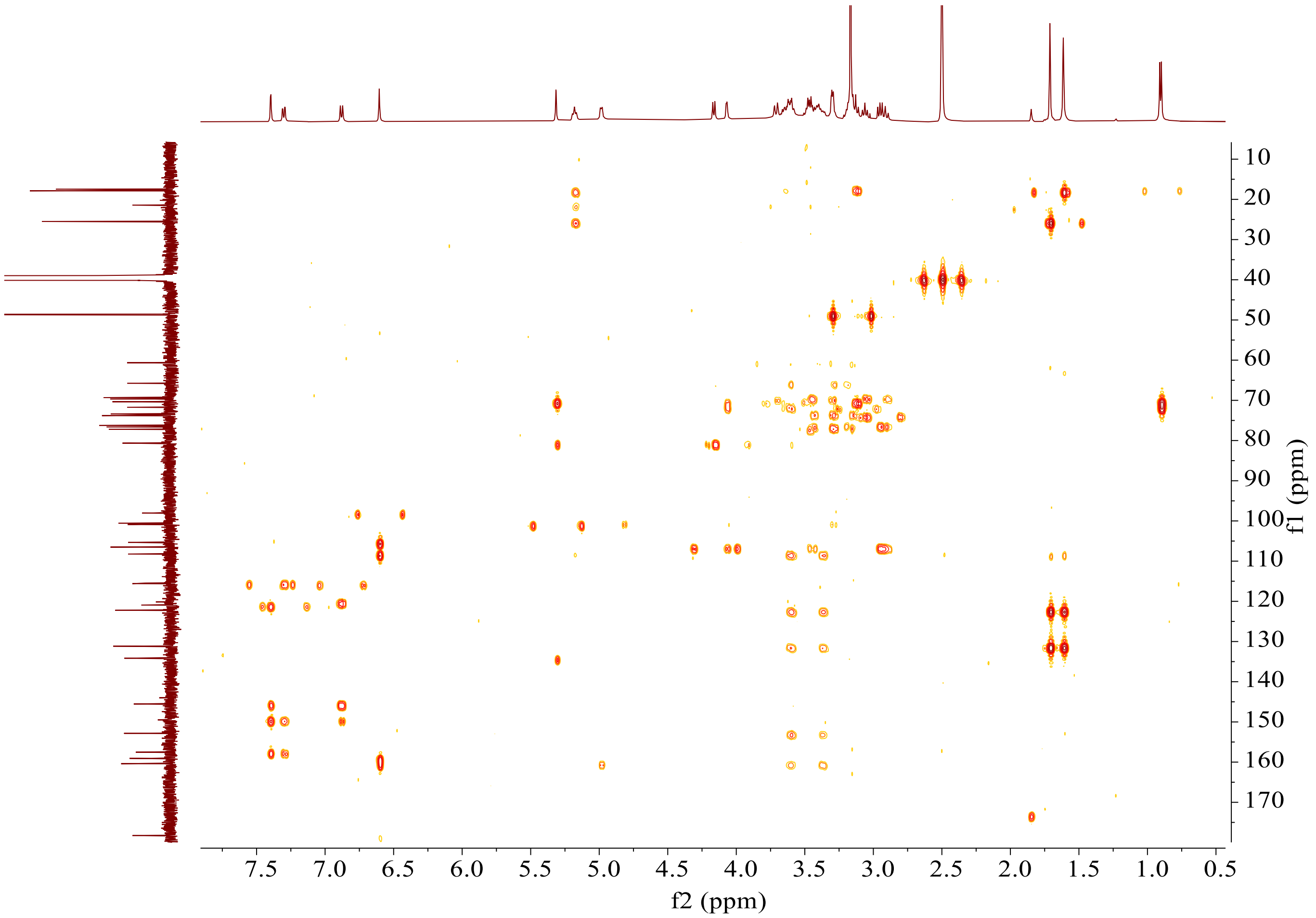

Figure S15. HMBC spectrum of compound **3**

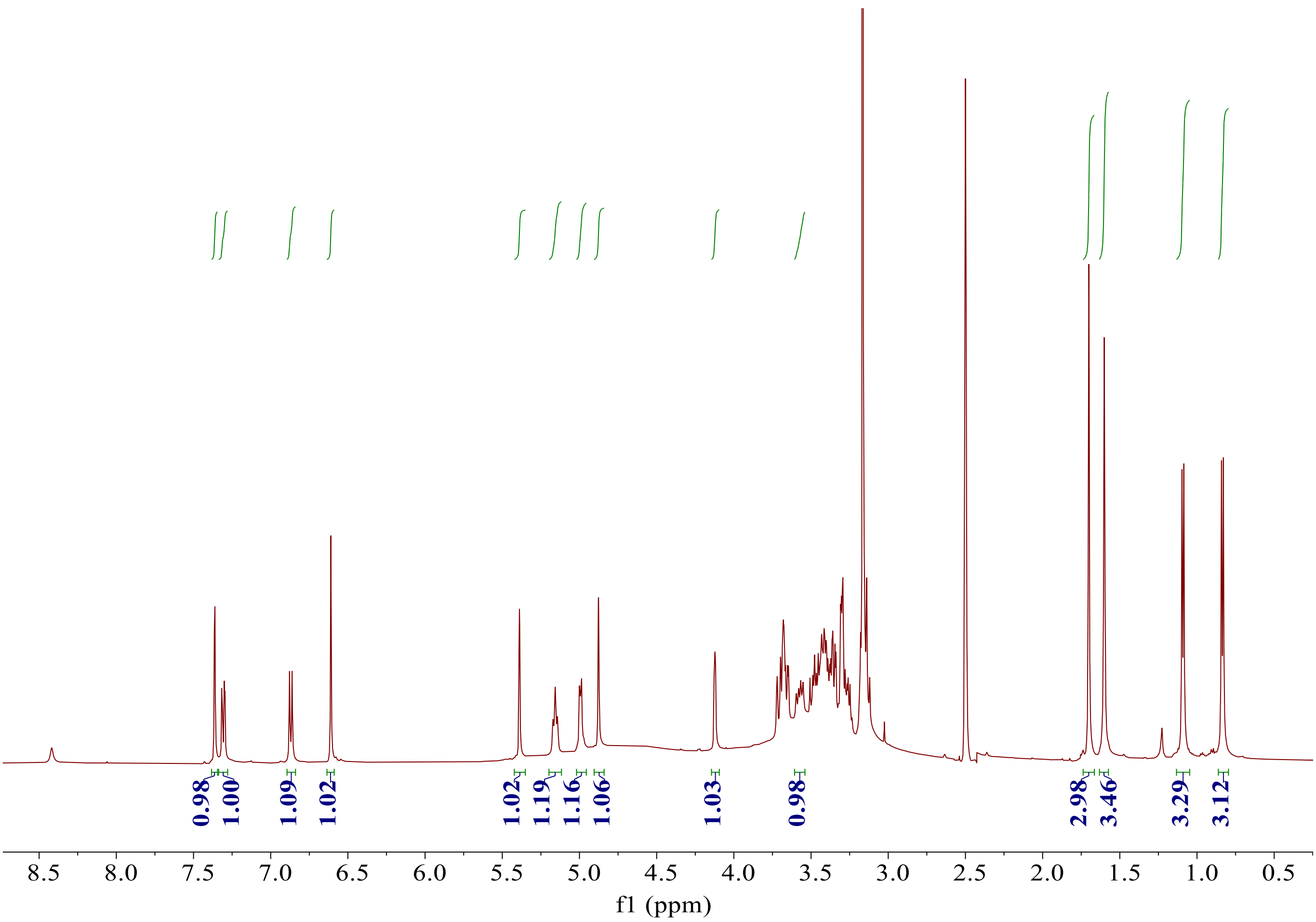

Figure S16. ^{1}H-NMR spectrum of compound **4** (500 MHz)

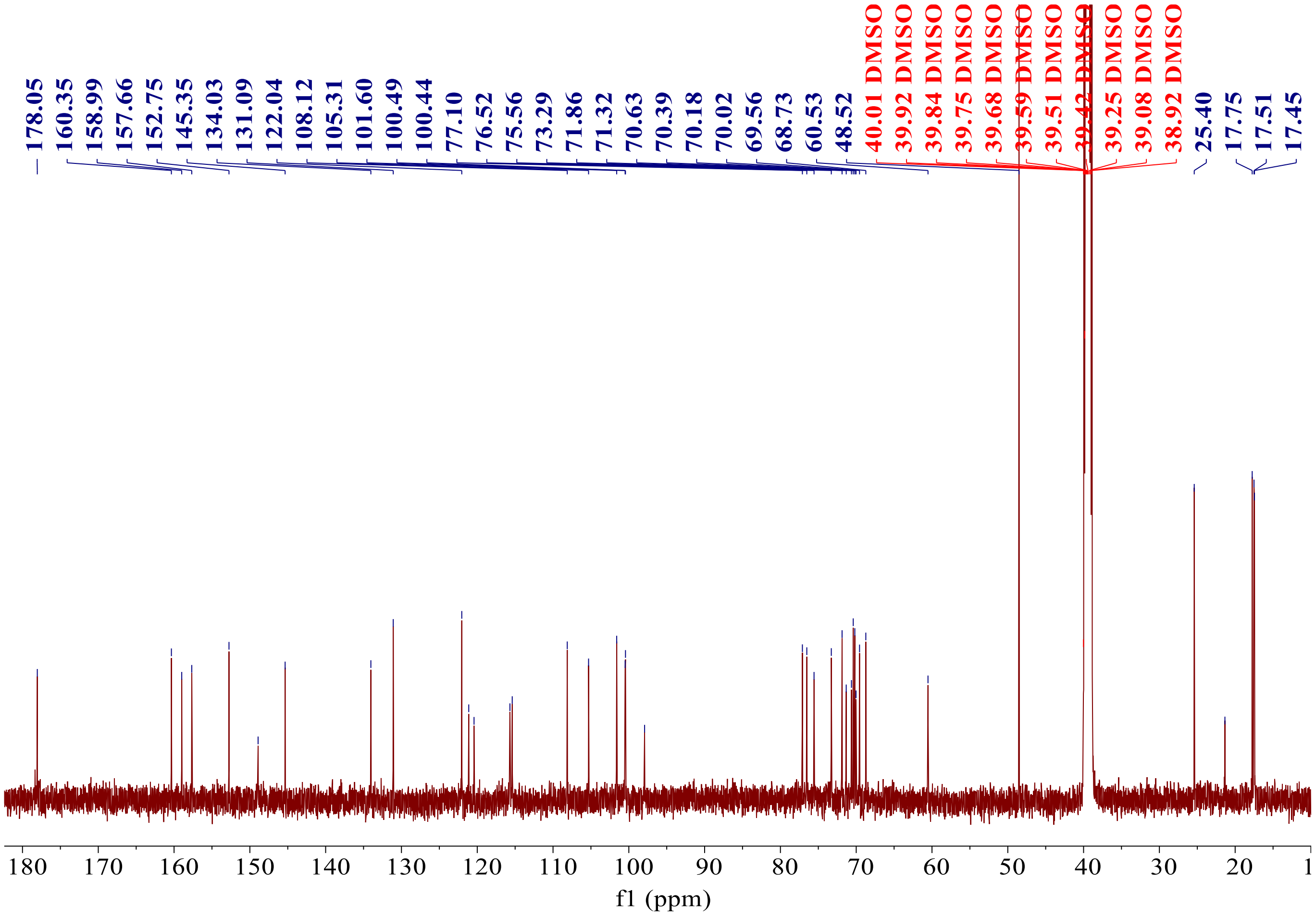

Figure S17. ^{13}C-NMR spectrum of compound **4** (125 MHz)

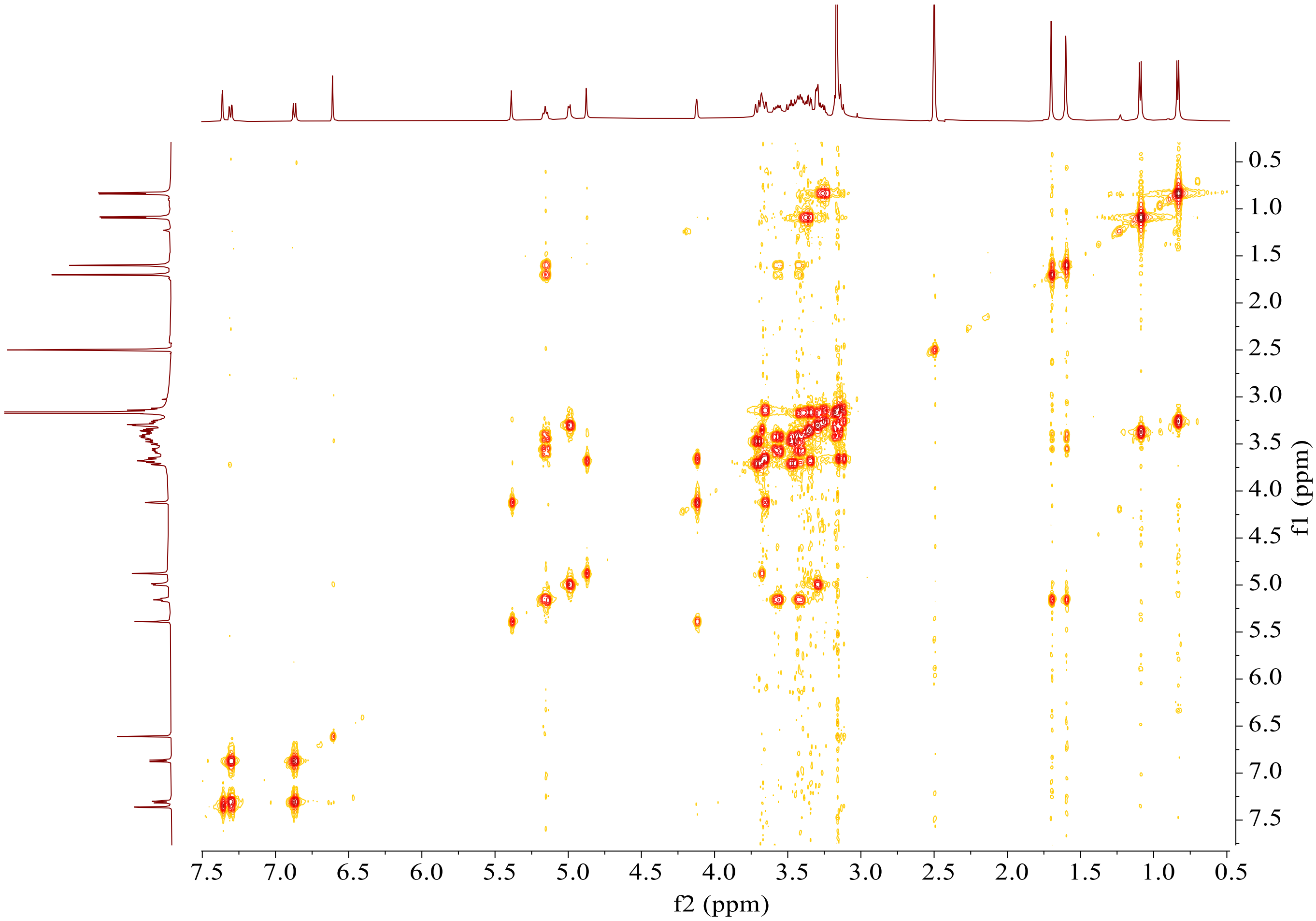

Figure S18. ^{1}H-^{1}H COSY spectrum of compound **4**

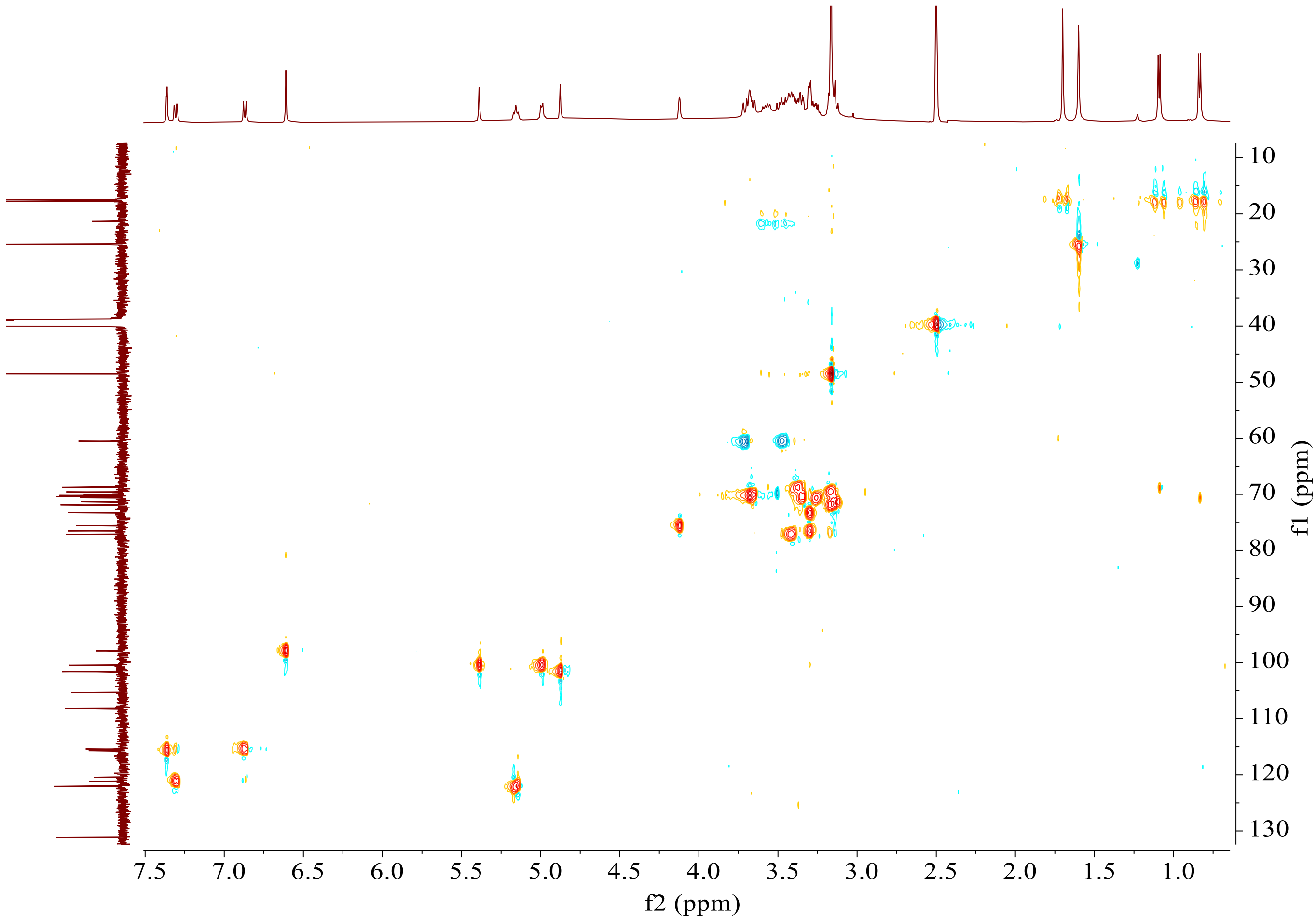

Figure S19. HSQC spectrum of compound **4**

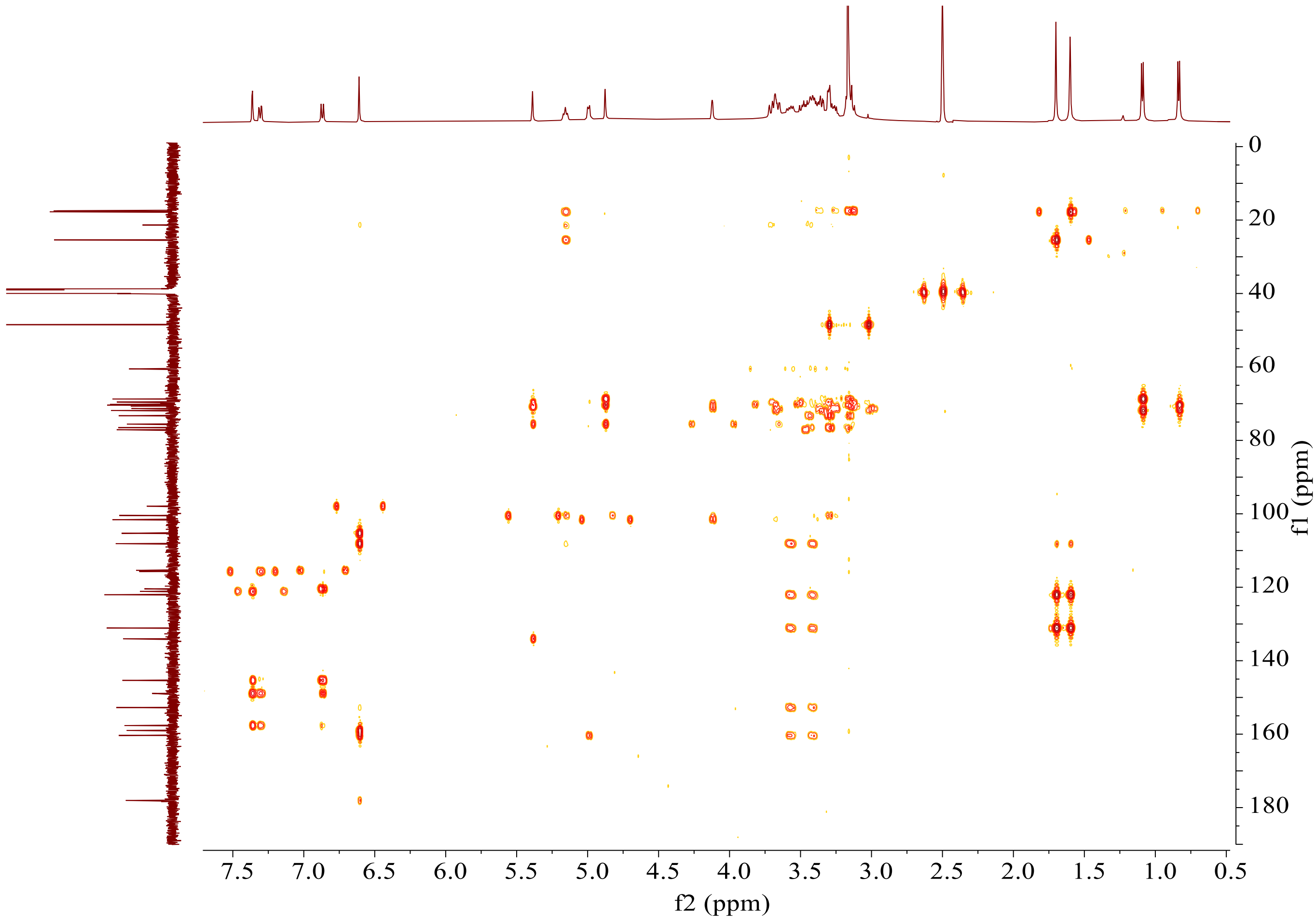

Figure S20. HMBC spectrum of compound **4**

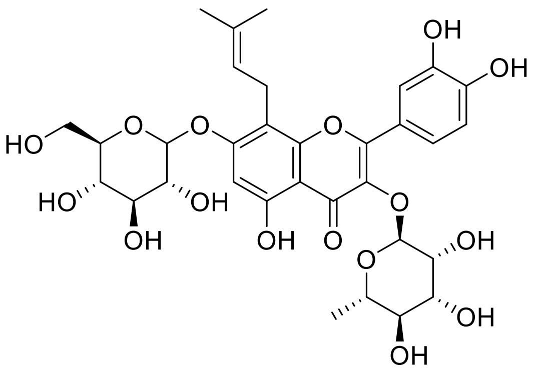

3'-hydroxyl-epimedoside A (5)

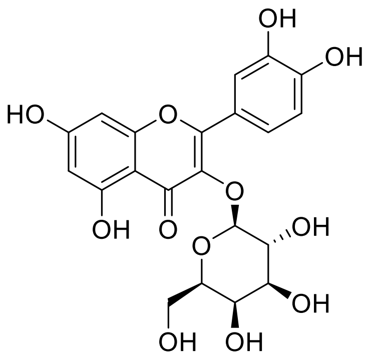

hyperoside (6)

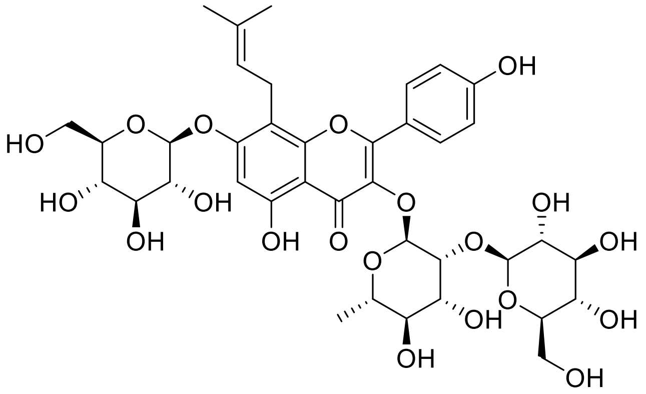

ikarisoside C (7)

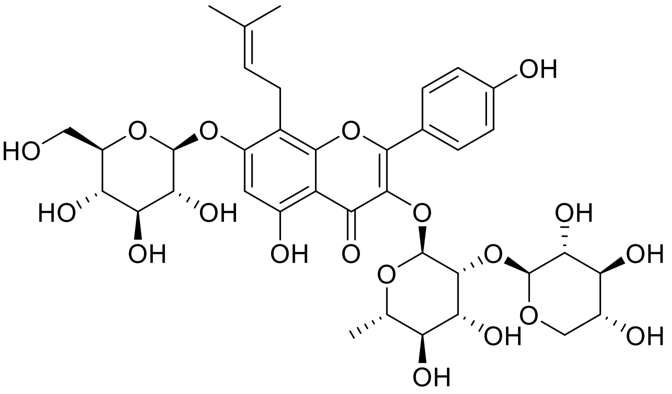

epimedoside E (8)

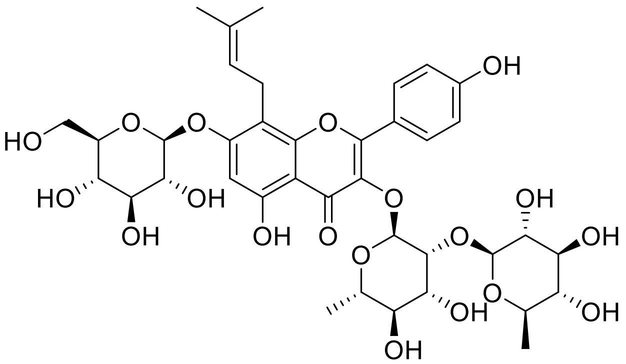

diphylloside B (9)

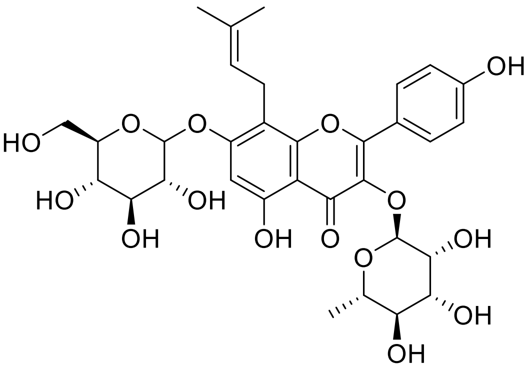

epimedoside A (10)

2. Chemical structures of compounds 5-10
